# Bio-electrosynthesis of polyhydroxybutyrate and surfactants in microbial fuel cells: a preliminary study

**DOI:** 10.3389/fmicb.2025.1372302

**Published:** 2025-02-25

**Authors:** Rosa Anna Nastro, Chandrasekhar Kuppam, Maria Toscanesi, Marco Trifuoggi, Andrea Pietrelli, Vincenzo Pasquale, Claudio Avignone-Rossa

**Affiliations:** ^1^Laboratory of Microbiology and Biochemistry, Department of Science and Technology, University of Naples “Parthenope”, Naples, Italy; ^2^Department of Biotechnology, Vignan’s Foundation for Science, Technology and Research, Guntur, Andhra Pradesh, India; ^3^Analitics Chemistry for the Environment Laboratory (ACE), CESMA, Department of Chemical Sciences, University of Naples “Federico II”, Naples, Italy; ^4^Laboratoire Ampere CNRS UMR 5005, Université de Lyon, UCBL, INSA, ECL, Villeurbanne, France; ^5^Laboratory of Systems Microbiology, Department of Microbial Sciences, University of Surrey, Guildford, United Kingdom

**Keywords:** *Cupravidus necator* DSM 428, PHBs electrosynthesis, *Pseudomonas aeruginosa* PA1430/CO1, *Shewanella oneidensis*-MR1, bioelectrochemical systems, CO_2_ capture, biosurfactants

## Abstract

Microbial Electrochemical Technology (MET) offers a promising avenue for CO_2_ utilization by leveraging the ability of chemolithotrophic microorganisms to use inorganic carbon in biosynthetic processes. By harnessing the power of electroactive bacteria, METs can facilitate the conversion of inorganic carbon into organic compounds. Therefore, this work combines biosurfactant production at the anode and PHB production at the cathode of Microbial Fuel Cells (MFCs), while testing the efficiency of Microbial Electrosynthesis Cells (MECs), and traditional culture in liquid media. This study employed a consortium of *Pseudomonas aeruginosa* PA1430/CO1 and *Shewanella oneidensis* MR-1, to provide reducing equivalents to *Cupriavidus necator* DSM428 for CO_2_ fixation and polyhydroxybutyrate (PHB) production. Glycerol was used as a carbon source by the anode consortium to investigate biosurfactant production. Additionally, Adaptive Laboratory Evolution (ALE) was employed to enhance the efficiency of this process to develop biofilms capable of synthesizing PHB from CO_2_ in MFCs under a controlled gas atmosphere (10% CO_2_, 10% O_2_, 2% H_2_, 78% N_2_). Observed results showed a higher direct CO_2_ removal from the gas mix in MECs (73%) than in MFCs (65%) compared to control cultures. Anionic (18.8 mg/L) and non-ionic (14.6 mg/L) surfactants were primarily present at the anodes of MFCs. Confocal microscope analysis revealed that the accumulation of PHBs in *C. necator* was significantly higher in MFCs (73% of cell volume) rather than in MECs (23%) and control cultures (40%). Further analyses on metabolites in the different systems are ongoing. Our data gave evidence that the anode consortium was able to provide enough electrons to sustain the chemolithotrophic growth of *C. necator* and the biosynthesis of PHBs at the cathode of MFCs, in a mechanism suggestive of the direct interspecies electron transfer (DIET), naturally occurring in natural environment.

## Introduction

The need for new green processes for the biosynthesis of value-added compounds has driven scientific research toward electrosynthesis and other bioprocesses. Among them, the bioelectrochemical system (BES) is a hybrid technology that combines microbiology and electrochemistry, providing a sustainable and efficient approach to address the pressing issues of climate change and environmental pollution (Ramírez-Vargas et al., 2018; Nastro et al., 2023). BES offers a promising approach to transforming CO_2_ into value-added multi-carbon compounds. BES can reduce CO_2_ electrochemically by harnessing the power of electrotrophic microorganisms providing the required energy input to drive CO_2_ fixation in the biocathode (Cabrera et al., 2021; Naderi et al., 2023; Temirbekova et al., 2023; Wu et al., 2023). This process, often called Microbial Electrosynthesis (MES), enables the biosynthesis of several organic compounds, (such as ethanol, acetate, formate, propionate, and isopropanol) (Kronenberg et al., 2017; Alvarez Chavez et al., 2022) by applying an external electric potential (<2.5 volts). The energy provided is the primary mechanism to reduce the thermodynamic stability of CO_2_, via pathways like the Wood-Ljungdahl pathway in *Clostridium* spp. (Yang et al., 2022). In recent years, MES has developed as a promising technique for the biosynthesis of polyhydroxyalkanoates (PHAs), which are polyesters classified as follows by their carbon side chain length: short-chain (3–5 carbons), medium-chain (6–14 carbons), and long-chain (14–25 carbons). Their general formula is [—O—CRH—CH_2_—CO—]n, where R denotes the side chain (e.g., methyl group for 3-hydroxybutyric acid (HB) or ethyl group for 3-hydroxyvaleric acid (HV)). Poly(3-hydroxybutyrate) (P(3HB)) is a prominent example of a short-chain PHA (Acharjee et al., 2022; Fu et al., 2023). The interest in PHAs in general, and PHBs in particular, lies in their potential industrial applications as a replacement for fossil fuel-based materials (Ranaivoarisoa et al., 2019). These bio-based polyesters are widely used in bioplastics, chemical additives, medicine, agriculture, wastewater treatment, and cosmetics. Besides CO_2_, PHAs can also be produced from carbon-rich waste substrates (Javaid et al., 2020; Shin et al., 2021). From a biochemical perspective, PHAs are energy-storage molecules synthesized by various microorganisms under stress conditions (limited essential nutrients) with excess carbon sources (Saravanan et al., 2022). The PAHs are a convincing alternative to synthetic polymers, demonstrating adequate mechanical properties similar to polypropylene and being thermoformable into diverse bio-based products, which directly reduce the detrimental impact of humans on the environment. The *Cupriavidus necator* H16 is a well-established microorganism that produces PHBs at an industrial scale (Al Rowaihi et al., 2018; de Melo et al., 2023). Nevertheless, the high production costs, 4–10 times greater than those of fossil-fuel-derived polymers (Saravanan et al., 2022; de Melo et al., 2023), hinder its widespread adoption. The integration of biopolymers into the global market can be facilitated through a thorough cost analysis and identification of technologies capable of reducing production costs while minimizing environmental impact, and BESs have the potential to fulfill both requirements and contribute to taking PHAs on the market in the future (Alvarez Chavez et al., 2022).

Since the pioneering work of Srikanth et al. (2012) demonstrated PHA biosynthesis at the cathode of Microbial Electrosynthesis Cells (MECs) using a consortium of microorganisms, research on electrochemically-driven PHA production has gained traction. Recent studies (Alkotaini et al., 2018; Lai and Lan, 2021; Shin et al., 2021; Pillot et al., 2022) various strategies regarding biosynthesis of PHAs in BES, including syngas utilization by *Cupriavidus necator* (Langsdorf et al., 2024), highlighted the potential of BESs for CO_2_ capture and PHAs production. Parallelly, BESs demonstrate promise in producing platform chemicals like biosurfactants at the anode (Pasternak et al., 2023; de Rosset et al., 2024). Biosurfactants are amphiphilic surface-active molecules due to their hydrophobic and hydrophilic moieties and they find applications in oil processing, the pharmaceutical sector (moisturizers, creams, and medicines), food (as emulsifiers), medical (antimicrobial agents), agricultural (fertilizers), and civil (waste and sewage treatment) industries. (Nikolova and Gutierrez, 2021; Sarubbo et al., 2022; Vigil et al., 2024). Among the substrates that can be used to produce biosurfactants, glycerol, a significant byproduct of biodiesel production, is a desirable substrate (Monteiro et al., 2018; Liu et al., 2022). Its utilization in MES for biosurfactant production (e.g., rhamnolipids from *Pseudomonas aeruginosa* (Bezerra et al., 2019; Zhao et al., 2021)) offers economic and environmental benefits by adding value to a waste product. Glycerol, a byproduct that can constitute up to 10% of total biodiesel production, necessitates well-organized management to lessen the overall cost of the biodiesel production process. This byproduct has several uses in different industries, such as chemical, textile, pharmaceutical, and food sectors. Additionally, it can be utilized to produce value-added compounds such as ethanol, hydrogen, propanoic acid, butanol, citric acid, and polyunsaturated fatty acids, primarily through microbial metabolism (Chilakamarry et al., 2021), and, as previously said, biosurfactants. The ability of *Pseudomonas aeruginosa* to produce rhamnolipids, a class of glycolipids, as metabolites of glycerol degradation in both aerobic and anaerobic conditions and the possible use of crude glycerol as a substrate for rhamnolipids production is very attractive from an industrial perspective (Bezerra et al., 2019; Zhao et al., 2021). Furthermore, recent studies have identified biosurfactant production, specifically glycolipids, in environmental isolates of the genus *Shewanella*. Nevertheless, the metabolic pathway for glycolipids biosynthesis in *Shewanella* remains to be clarified (Joe et al., 2019; Ram et al., 2019; Hassanshahian et al., 2020).

Several approaches, aiming at emulating the natural environment and gaining access to the untapped natural resources emerging from crosstalk between partners, have been widely employed in biomanufacturing to produce pharmaceuticals, nutraceuticals, food, and drinks on a large scale and play a prominent role in the bioremediation and bioenergy sectors (Kapoore et al., 2022). Co-culturing experiments involving the direct mixing of microorganisms have been proven to enhance microbial functions and accomplish tasks that are difficult to achieve with monocultures. Instead, exopolysaccharides (EPS) immobilize cells in biofilms by providing mechanical stability and keeping them close. As a result, in biofilm, an inter-connected cohesive three-dimensional polymer network is realized, where crosstalk between cells forms synergistic micro-consortia (Kapoore et al., 2022), in our case, a dual-species consortium. Starting from this assumption, we cocultured, since the very beginning of our experiment, *P. aeruginosa* and *S. oneidensis* firstly in liquid media and then in biofilms, to possibly enhance their interactions with potential advantages in terms of electron transfer to the anode and glycerol fermentation.

The integration of CO₂ capture at the cathode with surfactant biosynthesis at the anode in BESs leads to several advantages, including high operational efficiency, reduced production costs, lower energy consumption, and mitigation of environmental impacts (Jourdin et al., 2018; Gong et al., 2020; Ayol et al., 2021; Li and An, 2022). Hence, this integration approach provides a sustainable solution to climate change and pollution challenges (Hao et al., 2022). Electrosynthesis in MECs involves applying an electrochemical potential to control microbial metabolism or sustain abiotic electrochemical processes (Dattatraya Saratale et al., 2022). Alternatively, MFCs can generate reducing equivalents via electrogenic bacteria at the anode to support chemolithotrophic growth at the cathode. These systems utilize CO₂ with H₂ (or electrons and protons) to produce metabolites, providing a stable dynamic equilibrium amid the anode and cathode compartments (Nastro et al., 2023). Unlike what happens in MECs, MFCs work properly if the sum of the Gibbs Energy of the bioelectrochemical processes occurring at the anode and the cathode is negative [ΔG_tot_ = (ΔG anode + ΔG cathode) <0], leading to a self-sustaining system (Abdulwahhab et al., 2023; Nastro et al., 2023). Direct Interspecies Electron Transfer (DIET) is a key mechanism enhancing electron flow among microbial populations. This process, mediated by conductive materials (e.g., biochar), conductive pili, cytochromes, or molecular shuttles, enables the syntrophic growth of exoelectrogenic and electrotrophic bacteria, as seen in *Geobacter metallireducens* and *Methanosarcina barkeri* (Holmes et al., 2018; Chen et al., 2022; Valentin et al., 2023). In *C. necator*, conductive pili may enhance DIET efficiency by increasing cell-electrode contact distances and enabling more cells to participate in electron transfer. Their expression can significantly improve MFC performance by optimizing metabolic activity and power generation, paving the way for advanced bioelectronic materials and integration of such systems into bioelectronics applications. Starting from this information, we set up MFCs using a consortium of highly electroactive bacteria to verify whether such bacteria, once cocultured for several hundreds of generations, might be able to satisfy the demand of electrons of *C. necator* DM428 and, therefore, sustain its chemolithotrophic growth. *C. necator* utilizes the Calvin-Benson-Bassham Cycle for the assimilative reduction of CO_2_, facilitating the biosynthesis of glyceraldehyde-3-phosphate. In this process, H₂ molecules serve as electron donors for the O_2_-tolerant [NiFe]-hydrogenases, while O₂ acts as the terminal electron acceptor (Teramoto et al., 2022).

We hypothesized that in MFCs, in the absence of an external source of electrons, a mechanism like DIET might be established between the anode consortium and *C. necator*, thus fostering the syntrophic growth of bacteria at the anode and cathode, with concurrent biosynthesis of biosurfactants (at the anode) and CO_2_ capture at the cathode, with concurrent synthesis of potential added-value compounds. To test this, in this study, we aimed to investigate PHB production in *C. necator* DSM428 cells by applying different conditions: the presence of an external potential of −955 mV (*v*s Ag/AgCl reference electrode in MECs), of the electrons provided by *P. aeruginosa/S. oneidensis* consortium in MFCs, without electrogenesis in microbiological cultures in liquid medium. We drastically reduced the start-up period of BESs by using already mature biofilms at both anodes and cathodes, and microorganisms progressively grown in oligotrophic conditions. Once we set up the first BESs and control cultures, we repeatedly used strains isolated from anolytes and catholytes of BESs at the end of a cycle to start a new experimental session according to an Adaptive Laboratory Evolution (ALE) approach. ALE is a technique for the selection of strains with better phenotypes by long-term culture under a specific selection pressure or growth environment (Wang et al., 2023). Adaptive laboratory evolution (ALE) is the process based on which the principles of natural evolution are implemented in the laboratory to a specific population under controlled conditions. In ALE, natural selection is directed toward a selected environment with the desired conditions for microbial populations to obtain better fitness to improve one or more metabolic abilities of a given microorganism. Further, ALE is used as a tool for a deeper understanding of the genetic and/or metabolic pathways of evolution and, also, to improve the biosynthesis of products of (high) added value, such as ethanol, butanol, and lipids (Mavrommati et al., 2022). Following this approach, we also investigated the metabolic profile and intracellular PHB content in *C. necator* when used for several thousands of generations in MFCs. The obtained profile was compared with the original strain purchased at DSMZ, which had never grown in chemolithotrophic conditions. To our knowledge, this is the first time such an experimental scheme has been applied to *C. necator*.

## Materials and methods

### Microbial cultures protocols

The protocols for the preparation of the strains envisaged the gradual adaptation of the chosen microorganisms toward the growth in mineral media and the use of inorganic carbon (*Cupavidus necator*) and glycerol (*P. aeruginosa* and *S. oneidensis*) as carbon sources. More specifically, we suspended a lyophilized culture of *Cupravidus necator* DSM 428 in 10 mL of Nutrient Broth (Oxoid ©) and prepared cultures in both NB and Nutrient Agar (NA), according to the supplier’s instructions. All media were aerobically incubated at 30°C for 24 h. Subcultures were prepared in Tryptone Soy Agar (TSA), and incubated at 30°C for 48 h. We maintained part of the cultures on TSA for use in later experiments. We used well-isolated colonies to prepare 0.1 OD_600nm_ microbial suspensions in NaCl 0.9% saline solution, inoculated it in Tryptic Soy Broth (TSB) and incubated in shaking conditions at 30°C for 24 h. Then, we transferred 1 mL of culture in 9 mL of DSMZ 81 mineral medium and, at the end of the incubation, we repeated the procedure using the same fresh medium. The composition of DSMZ81 was: KH_2_PO_4_, 2.300 g, Na_2_HPO_4_ × 2 H_2_O 2.900 g, NH_4_Cl 1.000 g, MgSO_4_ × 7 H_2_O 0.500 g, CaCl_2_ × 2 H_2_O 0.010 g, MnCl_2_ × 4 H_2_O 0.005 g, NaVO_3_ × H_2_O 0.005 g, trace element sol. SL-6 (DSMZ medium 27) 5.0 mL, distilled water 915.0 mL. We added NaHCO_3_ 0.5 g/L as a source of carbon to prepare the biocathodes and *C. necator* control cultures. The pH was adjusted to 6.8 ± 0.2.

We started the experimental activity by culturing the single anodophilic strains *Shewanella oneidensis* MR1 (ATCC 700550) and *Pseudomonas aeruginosa* PA1430/CO1 (DSM 19882) in NB (according to the supplier’s instructions) and incubating them at 30°C for 24 h. We used TSA (pH 7.2 ± 0.2) to subculture both strains and maintain them for use in later experiments. Cocultures were prepared by adding 1 mL of *P. aeruginosa* PA1430/CO1 and 1 mL *S. oneidensis* MR-1 suspensions in 0.9% NaCl solution (both 1 OD_600_) to 18 mL of Tryptic Soy Broth (TSB) fresh medium and incubating them at 30°C for 24 h. At the end of the incubation, 2 mL were transferred to 18 mL of M9 Medium containing glycerol (4%) as the sole source of carbon and further incubated for 24 h at 30°C in a fresh medium. This last step was repeated twice before proceeding to the preparation of the bioanodes and the control cocultures (Nastro et al., 2023). At the same time control cultures, we transferred fresh and well-isolated colonies in TSB, and, subsequently, in M9 Medium containing glycerol (4%) as the sole source of carbon to prepare the cocultures as previously described. One liter of M9 contained: glycerol 40 mL, MgSO_4_ 1 mM, CaCl_2_ 0.3 mM, biotin 1 μg, thiamin 1 μg, 10 mL of trace elements solution (100X), 100 mL of salt solution (10X). The final pH was 7.2 ± 0.2. We assessed the presence and viability of both strains by spreading 100 μL of microbial suspension dilutions (from 10^−3^ to 10^−6^) on Pseudomonas Isolation Agar (PSI) and TSA. All media used in this research were purchased from Oxoid©. Glycerol, a biodiesel byproduct, is an ideal substrate for biosurfactant production in MES [e.g., rhamnolipids from *Pseudomonas aeruginosa*; (Bezerra et al., 2019; Zhao et al., 2021)] offering economic and environmental benefits by transforming waste into value-added products.

We started the experimental activity by culturing the single anodophilic strains *Shewanella oneidensis* MR1 (ATCC 700550) and *Pseudomonas aeruginosa* PA1430/CO1 (DSM 19882) in NB (according to the supplier’s instructions) and incubating them at 30°C for 24 h. We used TSA (pH 7.2 ± 0.2) to subculture both strains and maintain them for use in later experiments. Cocultures were prepared by adding 1 mL of *P. aeruginosa* PA1430/CO1 and 1 mL *S. oneidensis* MR-1 suspensions in 0.9% NaCl solution (both 1 OD_600_) to 18 mL of Tryptic Soy Broth (TSB) fresh medium and incubating them at 30°C for 24 h. At the end of the incubation, 2 mL were transferred to 18 mL of M9 Medium containing glycerol (4%) as the sole carbon source and further incubated for 24 h at 30°C in a fresh medium. This last step was repeated twice before preparing the bioanodes and the control cocultures (Nastro et al., 2023). In comparison to Pseudomonas Isolation Agar (PIA - used for the isolation of *P. aeruginosa* from water), we doubled the amount of glycerol to sustain *P. aeruginosa* and, possibly *S. oneidensis* metabolism, in absence of other carbon sources such as the peptic digest of animal tissue in PIA. At the same time control cultures, we transferred fresh and well-isolated colonies in TSB, and, subsequently, in M9 Medium containing glycerol (4%) as the sole carbon source to prepare the cocultures as previously described. One liter of M9 contained: glycerol 40 mL, MgSO_4_ 1 mM, CaCl_2_ 0.3 mM, biotin 1 μg, thiamin 1 μg, 10 mL of trace elements solution (100X), 100 mL of salt solution (10X). The final pH was 7.2 ± 0.2. We assessed the presence and viability of both strains by spreading 100 μL of microbial suspension dilutions (from 10^−3^ to 10^−6^) on Pseudomonas Isolation Agar (PSI) and TSA. All media used in this research were purchased from Oxoid©.

### Bioanode and biocathode assembly

Electrode material selection is critical in microbial fuel cells (MFCs). At the anode, it influences bacterial adhesion and biofilm growth, while at the cathode, it impacts redox reactions and power generation efficiency (Minutillo et al., 2020). Electrodes should exhibit high conductivity, large surface area, porosity, biocompatibility, and affordability. Carbon-based materials are ideal due to their microbial culture stability, electrical conductivity, biocompatibility, chemical stability, and corrosion resistance (Fan et al., 2021; Gambino et al., 2021). Carbon cloth’s graphite-like atomic structure and high conductivity facilitate electron transfer with microbial biofilms (Alarifi, 2019). This study used two carbon cloth anodes (NCBE, University of Reading, UK) with dimensions of 6.5 cm × 9 cm (projected area 58.5 cm^2^) and 1 mm thickness. The electrodes were folded once by threading with a wire Ni/Cr 80/20 wire (0.5 mm diameter - RS Components, Corby, UK), were sterilized with 70% ethanol, and assembled in 250 mL bottles, connected via a 100 *Ω* resistor, as per Nastro et al. (2023). The systems were completed by a sterile graphite rod (0.5 cm diameter, 7 cm length), partially submerged into the microbial suspension, and partially exposed to the air as a “snorkel electrode,” which, according to Hoareau et al. (2019) can increase the bioelectrochemical processes occurring at the electrode, thus fostering both the metabolism and electroactivity of bacteria in the anolyte of single-chamber MFCs. Nevertheless, no connection was established between the graphite rod electrode and the two carbon cloth electrodes intended to be used as anodes in our MECs and MFCs for CO_2_ capture and surfactant biosynthesis. Biofilm formation, as well as control culture growth, was carried out under shaking conditions (150 rpm/min) (Nastro et al., 2023). Further details are available in the supporting information file. We prepared 248 mL of TSB and added 2 mL of a 24-h coculture of the anode strains in TSB as described in the “Microbial cultures protocols section.” After 24 h of incubation at 30°C, we transferred the anodes (already colonized by *P. aeruginosa* and *S. oneidensis*) to fresh M9 (plus glycerol), and incubated at the same conditions for 24 h before being moved, again, to fresh M9 medium. After a further 24 h, the electrodes were placed in the anode chamber of the MFCs and MECs. The same approach was used to prepare the biocathodes colonized by *C. necato r*DM428. In this case, we first cultured *C. necator* in TSB and transferred the carbon cloth electrodes in DSMZ 81 medium, with NaHCO_3_ 0.5%, and aerobically incubated at 30°C for 24 h. This last step was repeated twice before the biocathodes were moved to the cathode compartment of the BESs. At every electrode transfer, we rinsed the electrodes with a sterile 0.9% NaCl solution to remove cells weakly linked to the biofilm EPS matrix. Before starting the CO_2_ capture experiment, we incubated the *C. necator* control cultures and BESs catholyte under an atmosphere of 10% (v/v) O_2_, 10% CO_2_, 2% H_2_, and 78% N_2_ for 8 h. The same gas mix was used during the CO_2_ capture experiment. Based on established literature, the CO₂ concentration was fixed at 10%, optimizing *C. necator* growth for polyhydroxyalkanoate accumulation as a carbon storage material (Lambauer and Kratzer, 2022; Chen et al., 2023; Lambauer et al., 2023).

### Set-up of two chamber-BESs

The experimental setup used during the direct capture of CO_2_ from gas mix consisted of 2-chamber MFCs and MECs, made with an acrylic body, with a volume chamber of 125 mL, where the preformed biocathode and bioanodes were placed. The chambers were separated by a cation exchange membrane (Fumatec FKE-50), with a double layer of polyester tissue (J-cloth, 0.2 mm thickness) holding the membrane to reduce biofouling. Both cell bodies and electrode material were purchased at (NCBE, University of Reading, Reading, UK). As previously stated, we used, respectively, M9 medium containing 4% glycerol and M81 medium added with NaHCO_3_ 0.5% final concentration, as anolyte and catholyte. After 24 h of operation, we replaced the NaHCO_3_ with CO_2_ from the gas mix (10% CO_2_). During the tests, the MFCs were connected to a 1,000 *Ω* external resistor, and the MECs were connected to a 1.5 V external battery (−955 mV at the cathode and + 545 mV at the anode,) All electrochemical potential values are reported, in this paper, *vs* Ag/AgCl, unless otherwise stated. The experiment was carried out twice, with systems prepared in double replicas. We also set up abiotic controls named MFC_blank, MEC_blank and sterile medium to evaluate the effects of the sole chemical–physical processes on CO_2_ capture from the gas mix.

**Figure 1:**
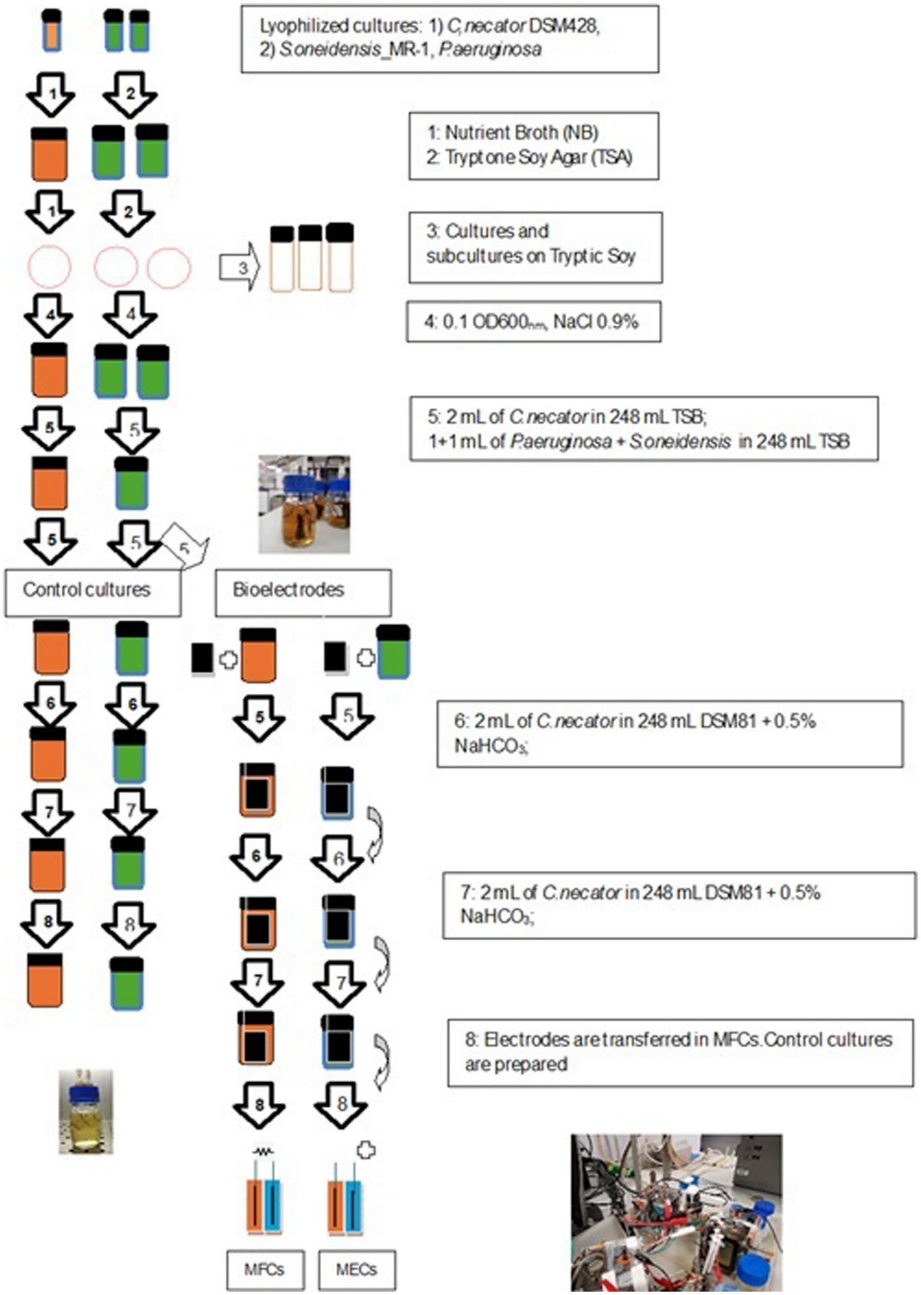
Schematic of the experimental procedure for both bioanodes and biocathodes preparation.

Once we collected the data, we started new experimental sessions using HCO_3_^−^ at the cathode. Even this time, we used two-chamber cells, with acrylic bodies but with an overall volume of 20 mL. Carbon cloth electrodes were 2.5 cm 3.5 cm (1 mm thickness), double folded with Ni/Cr 80/20 wire (0.5 mm diameter - RS Components, Corby, UK). Even in these systems, we used Fumatec FKE-50 cation exchange membrane and a double layer of J-cloth (0.2 mm thickness) on both sides of the membranes (Nastro et al., 2023). Both bioelectrodes were prepared using the same protocol reported in Figure 1, but we used 100 mL Duran bottle and 1 mL of microbial suspensions (0.5 OD_600_) diluted in 99 mL of medium to start both biofilms at the electrodes and control cultures. Once set up, the systems with already prepared bioelectrodes were left for one night in OCV and, after changing both anolyte and catholyte with fresh media, we connected, respectively, to the 1,000 *Ω* (MFCs) and − 1.5 V battery (MECs) at 20°C. All MECs, MFCs, and controls were set up in double replicates for three cycles of experimental activity, performed at 20°C, with each cycle lasting about three weeks, considering also the preparation of pre-colonized bioanodes and biocathodes and the performance of chemical analyses. Overall, BESs and control cultures operated for five days before being disassembled (Nastro et al., 2023). To assess the effect of preformed biofilms on MFCs behavior, we prepared two-chamber systems (20 mL overall volume) with sterile carbon cloth electrodes, directly inoculated with 0.5 OD_600_ (approx. 10^7^ UFC/mL) microbial suspension of the chosen strains at the anode and the cathode. We called these cells MFC_no_biofilm. Then, MFCs were left 24 h in OCV and incubated at 30°C. After that, we replaced the electrolytes and connected MFCs to 1,000 Ω. The systems were incubated for 24 h at 30°C before starting the tests in the presence of HCO_3_^−^ at the cathode. Even in this case, we prepared two replicas for each system and repeated the experiment three times. In all experiments, CO_2_ removal values obtained in BESs were compared to those observed in control cultures, where electrodes and energy inputs were unavailable.

### CO_2_ capture from the gas mix and inorganic carbon utilization

The CO_2_ capture experiment was carried out as described elsewhere (Nastro et al., 2023). Shortly, we sparged the selected gas mix in the cathode compartment of the BESs for 6 h under a pressure of 1.2 ± 0.2 atm. We replaced the DSMZ 81 medium with a fresh one and measured the CO_2_ concentration in the headspace, after 15 min of further gas mix sparging by connecting the cathode chamber exhaust tubing to the CO_2_/O_2_ meter (Electrolab CO_2_/O_2_ Off-Gas Analyser) (Electrolab Biotech Ltd., Tewkesbury, UK). We took catholyte, anolyte, and control culture samples for analysis. We calculated the CO_2_ capture rate (CO_2 capt_) from the gas mix according to Equation (1):


(1) $$CO_{2\mathrm{capt}}=\frac{\left[CO_{2\mathrm{in}}-CO_{2ev}\right]}{CO_{2\mathrm{in}}}x\;100$$

where CO_2capt_ is the percentage of CO_2_ subtracted from the gas mix sparged in the cathode compartment, CO_2in_ is the initial concentration of CO_2_ in the gas mix, and CO_2ev_ is the outlet amount from the chamber headspace. We also calculated the CO_2_ flow rate, crucial for maintaining optimal growth conditions for *C. necator*. We applied the mass conservation law for incompressible gases, the Bernoulli law for determining the gas flow velocity through a pipe, and calculated the molar flux of CO_2_ (expressed as mol/s) sparged in the DSM81 medium. We also calculated the CO_2_ concentration in the DSM 81 medium at saturation by applying the Henry’s Law equation for gas dissolution in water at 20°C. All formulas and calculations are reported in the Supporting Information section. If this approach was used to evaluate the ability of bacteria to remove CO_2_ from a gas mix continuously sparged in the catholyte as well as in a *C. necator* suspension, a more accurate evaluation of the inorganic carbon utilization by a microorganism requires the measure of the inorganic carbon (HCO_3_^−^) dissolved in the culture medium. For this reason, we used a CO_2_ probe, connected to an M400 Type 2 transmitter (iPro5000, Mettler Toledo, US) to evaluate the variation of dissolved CO_2_ over time.

The resulting concentrations were reported *vs* the amount of residual CO_2_ in control cultures, and expressed as percentages. We then repeated the experiment using DSM81 medium with NaHCO_3_ 0.5% at pH 6.8, corresponding approximately to 26 ± 2 mg/L of dissolved CO_2_ at the cathodes and in control cultures. The next step envisaged the set-up and monitoring of strains used in MFC (*C. necato*r_MFCs) and cells from the collection strain (*C. necator*_coll), with either preformed and not already established biocathodes. We set up all systems in double replicates, for three cycles of tests for HCO_3_^−^ utilization, at 20°C. All MECs, MFCs, and controls were set up in double replicates for three cycles of experimental activity, performed at 20°C, with each cycle lasting about three weeks, considering also the preparation of pre-colonized bioanodes and biocathodes and the performance of chemical analyses. Overall, BESs and control cultures operated for five days before being disassembled (Nastro et al., 2023).

The continuous transfer of bacteria from an “old” MFC to a newly assembled one resulted in the growth of both anode and cathode microorganism for several thousand generations in well-defined oligotrophic conditions and electrochemical potentials, with the anode consortium continuously stimulated (in MFCs) to degrade glycerol and transfer electrons to the anode while *C. necator* was grown in chemolithotrophic conditions receiving just the electrons from the anode as reducing equivalents to be transferred to the RUBISCO. The unavailability of organic compounds as carbon source and the exposition to a well-defined range of electrochemical potentials at the cathode might have induced metabolic adaptation (if not changes in the genome) and a modification in the pattern of carbon source that can be used to sustain the microbial growth. To verify our hypothesis, we set up MFCs inoculated with strains never used in previous experiments and evaluated both CO_2_/HCO_3_^−^ utilization in *C. necator*, biosurfactants production by Pseudomonas/Shewanella consortium, and MFCs electrochemical stability. We named these systems MFC_coll. Similarly, to investigate the effect of preformed biofilms on MFCs performance, we checked on the behavior of MFCs with sterile electrodes and inoculated with 0.5 OD_600_ (approx. 10^7^ UFC/mL) microbial suspension of the chosen strains at the anode and the cathode. We called these cells MFC_no_biofilm and evaluated the inorganic carbon utilization in the presence of HCO_3_^−^.

### Electrochemical analyses

We used an Arbin Fuel Cell Test Equipment to monitor the initial Open Circuit Voltage (OCV), current production and polarization behavior of MFCs. In each polarization experiment, we used 14 steps of 5 min, from 36 k*Ω*–100 Ω. Cyclic Voltammetry (CV) was carried out at cathodes of MECs and MFCs, previously sparged with CO_2_, in a range of −1 V to +1 V (scan rate of 1 mV/min). The tests were conducted at pH of 6.8 ± 0.2, with a PalmSense 4S potentiostat and Ag/AgCl as reference electrodes. At the end of the CO_2_ capture experiment, we collected the data recorded by the ARBIN Fuel Cell Test Equipment and, then, calculated the current and power density, the energy produced expressed, respectively, as mA/m^2^, mW/m^2^, mW/h, and referred to the cathode surface. From the measure of the current intensity, we estimated the number of electrons used by *C. necator* in MFC_1000Ω to remove one mole of CO_2_ from the gas mix by using the following formulas (Equations 2, 3):


(2) Q=i∗t

where *i* is the current flowing through the external circuit while the MFCs we connected to the 1,000 Ω external resistor and measured in Ampere (A), Q is the number of charges expressed as Coulomb (C) and t is the time in seconds (s). From the Q values, we calculated the mole of electrons (n _e-_) according to the following calculations:


(3) $$n_{e-}=\frac{Q}{F}$$

where F is the Faraday constant whose value is 96,485 C/mol. We estimated the n _e-_ used by MECs (we applied a 1.5 V external battery) according to Kirchhoff’s law (Nastro et al., 2023), reported in Equation (4):


(4) $$I_{b}=\left(V_{b}-OCV_{MEC}\right)/R_{MEC}$$

Where *I_b_* is the current intensity provided by the battery *b*, *OCV_MEC_* is the Open Circuit Voltage of MEC after being disconnected from the battery and achieving a constant voltage. *R_MEC_* is the internal resistance of MEC. The overall power provided to the cells was calculated as follows (Equation 5):


(5) $$P_{b}={V_{b}}^{*}I_{b}={V_{b}}^{*}\left[\left(V_{b}-OCV_{MEC}\right)/R_{MEC}\right]$$

Where 𝑃_𝑏_ and V*_b_* are, respectively, the electric power and the voltage provided by the batteries, I*_b_* is the current intensity flowing through the systems, OCV_MEC_ and R_MEC_ as above. Starting from P_b_ values, we calculated the current intensity (*i*_MEC_) provided during the CO_2_ assimilation experiment. From *i*_MEC_, we obtained the $n_{e-}$ as described for the MFCs.

### Screening of PHBs in *Cupriavidus necator* cells

Soon after finishing the experiments with CO_2_ gas mix and HCO_3_-, we took samples of catholytes, control cultures, and cathodes and immediately froze them at −20°C. After defrosting them, we used a rapid test with 96-well microplates as a high throughput means to measure the fluorescence intensity of the Nile red stained cells in catholytes and microbial cultures containing PHBs, according to Zuriani et al. (2013). This rapid test allowed a first evaluation of the cellular contents in *C. necator* grown at MECs, MFCs catholyte, and control cultures and the identification of the most suitable conditions (among the ones realized in the different systems) for PHBs. First, we prepared a 10 mg/mL stock solution and a 1 mg/mL working solution of Red Nile (Merck) in dimethylsulfoxide (DMSO, Sigma-Aldrich), and stored it at 5°C for no longer than one month. At the end of the direct CO_2_ capture experiments and of the following tests with HCO_3_^−^ cycle of experiments, we took 1 mL of catholyte of MFCs and MECs and control cultures and centrifuged them at 12000 g for 5 min at 5°C. After resuspending the pellet in 1 mL of sterile distilled water, we added 4 mL of the Red Nile working solution. All samples were prepared in double replicates. After 30 min of incubation at room temperature (20 ± 2°C), we centrifuged the samples at 12000 g for 10 min at 5°C. We discarded the supernatants and replaced them with distilled water. We resuspended the pellets and distributed 200 mL of each in 96-well Microplates for Fluorescence-based Assays (Thermo Fisher Scientific). We measured the emission at 598 nm (with 543 nm excitation wavelength) at a Clariostar BMG LABTECH multi-plate Reader, with an incubation temperature of 30°C. Each essay was performed in 3 replicates. Standard solutions of 0.002, 0.02, and 0.2 mg/mL of poly[(R)-3-hydroxybutyric acid] (Sigma-Aldrich), dissolved in DSMO were prepared and treated in the same way as the microbial samples. It has been demonstrated that PHB granules’ structure and position in the cells can change in different organisms and even in reason of the metabolic state of a given organism, although the reason why this happens is not clear (Bordel et al., 2021). Therefore, we investigated the intracellular distribution and the granules/cells volume ratio in cells grown in the absence of electrodes (control cultures), in the presence of an applied potential (MECs), and electrons delivered from the *Pseudomonas/Shewanella* anode consortium.

Samples of microbial suspensions and cathode fibers were prepared for confocal microscopy analyses with Red Nile (10 μg/mL in DMSO), carried out according to Juengert et al. (2018). Pieces of fibers were taken from the four sides of the cathodes by using sterile scissors, and placed in 5 mL vials, together with 4 mL of the Red Nile working solution. After 30 min of incubation at room temperature (20 ± 2°C), we discarded the stain and replaced it with distilled water to remove the excess of Red Nile. We then prepared agarose pads by pipetting 100 μL of hot (~60°C) agarose solution (1% [w/v] in H_2_O) on the slide, immediately placing the cover slip on the agarose and letting it solidify for at least 2 min without removing the cover slip. After that, we added 4 μL of cells from control cultures and catholytes, previously centrifuged 13,000 × *g* for 60 s and resuspended in 30 μL of 0.9% NaCl solution, to 1 μL of Nile red and waited 30 min before transferring 1 μL of stained cells on the agarose pads. As to the fibers, we used sterile forceps to move the fibers on the agarose pads. The same protocols were applied to all samples. The slides were observed at the microscope, a Nikon Ti-Eclipse microscope (MSc +) provided with Confocal 4 lasers (405 nm, 457–488-514 nm, 561 nm, 643 nm), widefield filters (Brightfield, DAPI, FITC, TRITC, Cy5), phase contrast and DIC on selected lenses. We analyzed the images using NIS-Elements AR 5.21.03 (Nikon) software. While observing the slides, we followed a key pattern (20 fields) at different magnification values (10x, 40x, 60x, and 100x), in the bright field first and, then, at an excitement wavelength of 545 nm, with 590 nm emission). We evaluated the intracellular distribution and estimated the volume of the granules reported to the cells volume, according to Mravec et al. (2016).

### Metabolic profile analyses

As previously stated, during the whole experimental activity, we used an ALE approach to investigate the behavior of the chosen microorganisms after continuous and prolonged use of MFCs. Starting from the preparation of preformed biofilm and during all cycles of experiments, once we ended an experimental session and before disassembling our systems, we isolated bacteria from both analytes and catholytes of MFCs as well as from control cultures. We have used them in further experiments, as previously described and also reported in Nastro et al. (2023). As a result, the original lyophilized strain was used to prepare just the biofilms and cultures for the first test for direct CO_2_ capture from the gas mix. We use ECOPLATE tests (BIOLOG) for the physiological profiling (CLPP) of *C. necator* at the beginning and at the end of the whole experimental activities and effectively distinguish any changes in its metabolism. BIOLOG ECO microplates contain 31 different carbon substrates that are classified into six categories: carbohydrates, carboxylic acids, amino acids, phenolic compounds, polymers, and amines/amides and, for this reason, it is particularly useful to assay Carbon Source Utilization Patterns (CSUP) for single strains and microbial communities, which can change in consequence to a change in the environmental conditions of their growth such as the exposition to a toxic compound or the availability of carbon sources (Martínez-Toledo et al., 2021). Briefly, after collecting the results of chemical analyses and BESs monitoring, we isolated *C. necator* from the cathode of one of the MFCs replica of the last round of experiments and prepared a suspension of 0.2 McFarland in NaCl 0.9% solution and inoculated it in an ECOLOG 96-multiwell plate (BIOLOG), sealed in a plastic bag with wet paper sheets, and incubated at 30°C for 72 h. The same procedure was followed for the strain from the original liophylized culture. We measured the absorbance of the ECOLOG plates after 24, 48,72 and 144 h at 590 nm by a Clariostar Multimode Microplate Reader (ThermoFisher). From the results from the Clariostar, we calculated the average well-color development (AWCD) as a measure of the overall metabolic activity of microorganisms, grown in the presence of each test substrate (Nastro et al., 2023).

### Biosurfactant production

The drop collapse essay assessed the production of surfactants, an indirect method that relates the water surface tension to the concentration of surfactants (Gambino et al., 2017). To detect the presence of biosurfactants in *Pseudomonas/Shewanella* co-cultures and BESs anolytes, we filtered both culture supernatants and anolytes by a sterile nitrocellulose filter (0.22 μm) and applied a drop collapse essay on a surface previously coated with mineral oil. We recorded the collapse by filming the drops using a Keyence VHX-5000 microscope, connected to a camera. The time that a single drop takes to collapse is a function of surfactant concentration (Supplementary Table S1 in the supporting materials section). This test alone does not provide information about the chemical nature of the biosurfactants nor give a proper quantification of their concentration in the medium, but it is used for quick preliminary screening (Gambino et al., 2017). For the determination of surfactants, including anionic, cationic, and non-ionic surfactants. These methods are based on the interaction between the surfactant molecules and specific reagents that result in the formation of colored complexes. Anionic surfactants typically include substances like sulfates, sulfonates, and carboxylates. One common colorimetric method for their determination is based on the reaction with Methylene Blue. The measurement wavelength is 606 nm. Cationic surfactants often contain ammonium or other positively charged groups. A common colorimetric method for their determination involves the formation of a colored complex with a dye like Bromophenol Blue, which is extracted in chloroform and evaluated photometrically. The nonionic surfactants test procedure measures alkylphenol ethoxylates (AP(EO)n), fatty alcohol ethoxylates (FA(EO)n), and polyethylene. Nonionic surfactants (ethoxylates with 3–20 ether bridges) react with the indicator TBPE to form colored complexes, which are extracted in dichloromethane. The measurement wavelength is 606 nm (Hach, DR 3900, Germany). Along with *C. necator*, we carried out an ALE experiment with the *Pseudomonas*/*Shewanella* consortium by continuously transferring the strains at the end of an experimental session from the anolyte of MFCs, MECs, and control cultures in newly assembled systems, as reported in the “CO_2_ capture from the gas mix and inorganic carbon utilization” section.

### Statistical analysis

We carried out a Principal Component Analysis (PCA) based on the correlation among CO_2_ removal from the gas mix, CO_2_ concentration in the catholyte and *C. necator* control cultures, PHBs concentration (mg/ml), VPHB/Vcell (%), anionic and non-ionic biosurfactants in anolytes and Pseudomonas/Shewannella cultures. We also carried out an Agglomerative Hierarchical Clustering Analysis (AHCA) based on Ward’s method and the calculation of Euclidean distance for dissimilarity. We used XLSTAT 2022 software (v. 24.4.1377—Addinsoft, Paris, Ile-de-France, France) to perform all the statistical analysis. All tests were carried out with a significant level *α* of 0.05.

## Results

### CO_2_ capture from the gas mix and inorganic carbon utilization

The application of the mass conservation and Bernoulli laws revealed a gas flow speed of 3.7 m/s and a molar flow of CO_2_ of 4.23E^−04^ mol/min corresponding, at 20°C and 1.2 atm, to 8.7 mL CO_2_/min, provided for 15 min for a total amount of 130.5 mL of the CO_2_ supplied at the cathode. By applying Henry’s law for the dissolution of a gas in diluted water solutions (<1 M), the concentration of CO_2_ at saturation at 20°C in the sole medium was 0.0044 mol/L (145 mgCO_2_/L), being the Henry’s constant for CO_2_ at 20°C equal to 0.037 molL^−1^ atm^−1^ and 0.12 atm the partial pressure of CO_2_ in the headspaces of our systems (see supporting information section).

The results of the direct CO_2_ capture with the 250 mL systems (125 mL cathode volume) are reported in Table 1. According to the direct measurements carried out during the gas mix sparging, the CO_2_ escaping from MECs and MFCs catholytes decreased by about 73 and 65% in MECs and MFCs, respectively, compared to the control cultures. The same trend was observed when we measured with the Metler Toledo CO_2_ probe the dissolved CO_2_ in the medium, approximately 5 min after finishing the gas sparging in all systems (MFCs, MECs, and control cultures). As to the abiotic controls, we found a decrease in CO_2_ concentration in the gas mix escaping from the systems of 8.5% in MFCs, 9% in MECs, and 21% in the sterile medium on average, consistent with what was observed in a similar experiment with *Clostridium saccharoperbutylacetonicum* N1-H4 and reported in Nastro et al. (2023). The decrease of CO_2_ due to abiotic processes in MFCs and MECs falls within the standard error, unless what was observed for the control culture where the effect of chemical–physical processes on CO_2_ accounted for approximately 1.1%. The meaning of this result is still to be ascertained. Since the values of dissolved CO_2_ decreased in time, we took two consecutive measures for each replica in a range of time not longer than 5 min, after stopping the gas mix sparging. Compared to the control cultures, we found a − 42.7% and a − 17.8% dissolved CO_2_ in the catholytes of MECS and MFCs, respectively (Table 1). At the end of the experiment, the concentrations of dissolved CO_2_ in the medium were 1.48 ± 0.06,

**Table 1:** Percentage of CO_2_ in the headspace of BESs, pH, and CO_2_ concentration values in catholytes and control cultures.

	MFC	MEC	Control
pH	6.8 ± 0.2	7.0 ± 0.2	6.2 ± 0.2
CO_2_% in the gas mix*	1.9 ± 0.3	1.4 ± 0.2	5.4 ± 0.4
Dissolved** CO_2_ (mg/L)	65.4 ± 2.6	45.6 ± 1.8	79.6 ± 2.3
Dissolved (CO_2_) mMol/L	1.48 ± 0.06	1.0 ± 0.04	1.81 ± 0.05

We report the average concentrations (double replicates for each system used in two cycles) ± standard error (SE). *10% CO_2_ initial concentration in the gas mix, **145 mg CO_2_/L in the sterile medium at saturation calculated by Henry’s Law.

1.0 ± 0.04 and 1.81 ± 0.05 mMol/L in MFCs, MECs, and control cultures, respectively. Therefore, MECs were more efficient in terms of CO_2_ assimilative reduction than MFCs.

The second part of our experimental activity, as previously stated, was aimed at assessing the influence of preformed biofilm, as well as previous metabolic adaptation, on MFCs performances using HCO_3_^−^ at the cathode. The results showed, for strains already acclimated to grow in MFCs under a 1,000 *Ω* external load, a removal efficiency of dissolved CO_2_ in MFCs of 60% at pH 6.8 ± 0.1 when grown in preformed biocathode, after 24 h of incubation at 20°C in DSM81 medium with 0.5% NaHCO_3._ Interestingly, when we directly inoculated *C. necator*_MFC and *C. necator*_coll in the cathode chamber with a sterile medium and carbon cloth electrode we obtained a decrease in CO_2_ concentration of 67 ± 3% and 76 ± 2%, respectively. A shift in the chemical equilibrium between CO_2_ and HCO_3_^−^ can also explain this last result as pH values were 7.2 and 7.6 in the presence of *C. necator*_MFC and *C. necator*_coll, both higher than the 6.8 ± 0.1 we detected in the MFCs_1000Ω, where we used preformed biofilms with strains undergone to the ALE. It is well known how the pH affects the equilibrium of inorganic carbon species (CO_3_^2−^, HCO_3_^−^, and CO_2_) in the water environment. Therefore, a proper evaluation of the effects of pH on CO_2_ dissolution needs to be taken into account and, at the same time, a proper investigation of *C. necator* metabolism in the different growth conditions is needed to explain this result.

### PHBs screening

The measure of the fluorescence intensity of Nile Red-stained *C. necator* cells in catholytes and microbial cultures showed, for microorganisms grown in the MECs cathode compartment and in control cultures, values below the instrumental threshold. On the contrary, we detected 0.013 ± .4E^−03^ mg PHBs in planktonic cells suspended in 1 mL of MFC_1000Ω catholyte. As the results of the first experimental session with gas mix suggested a higher CO_2_ utilization in MECs, but a very low production of PHBs, we started further tests with prolonged operation times of all systems by using DSMZ81 containing 0.5% NaHCO_3_ at the cathode and preformed bioanodes and biocathodes. At pH 6.8 and 20 ± 2°C, dissolved CO_2_ (measured with Mettler Toledo m400 CO_2_ probe) was 60 mg/L in the sterile medium. After an initial Open Circuit Voltage (OCV) 24 h, we applied to MECs an external voltage of 1.5 V (−955 mV and + 455 mV, respectively, at the cathode and the anode) for 16 h. We prepared control cultures and MFCs as well. Our results showed, the presence of 0.0122 ± .2E^−03^ mg/mL of PHBs in MECs catholyte (OD_600_ = 0.008), we measured 0.0088 ± 4.0E^−04^ mg/mL of PHBs in control cultures (OD_600_ = 0.004). In MFC_1000Ω catholyte (OD_600_ = 0.059), instead, we measured 0.028 ± 4.0E^−03^ mg/mL of PHBs after 24 h of OCV and 3 h of connection to an external resistor of 1,000 Ω. For the whole time, MFCs produced a stable voltage of 4.9 ± 1.1 mV, and 0.115 mW/h of energy.

**Figure 2:**
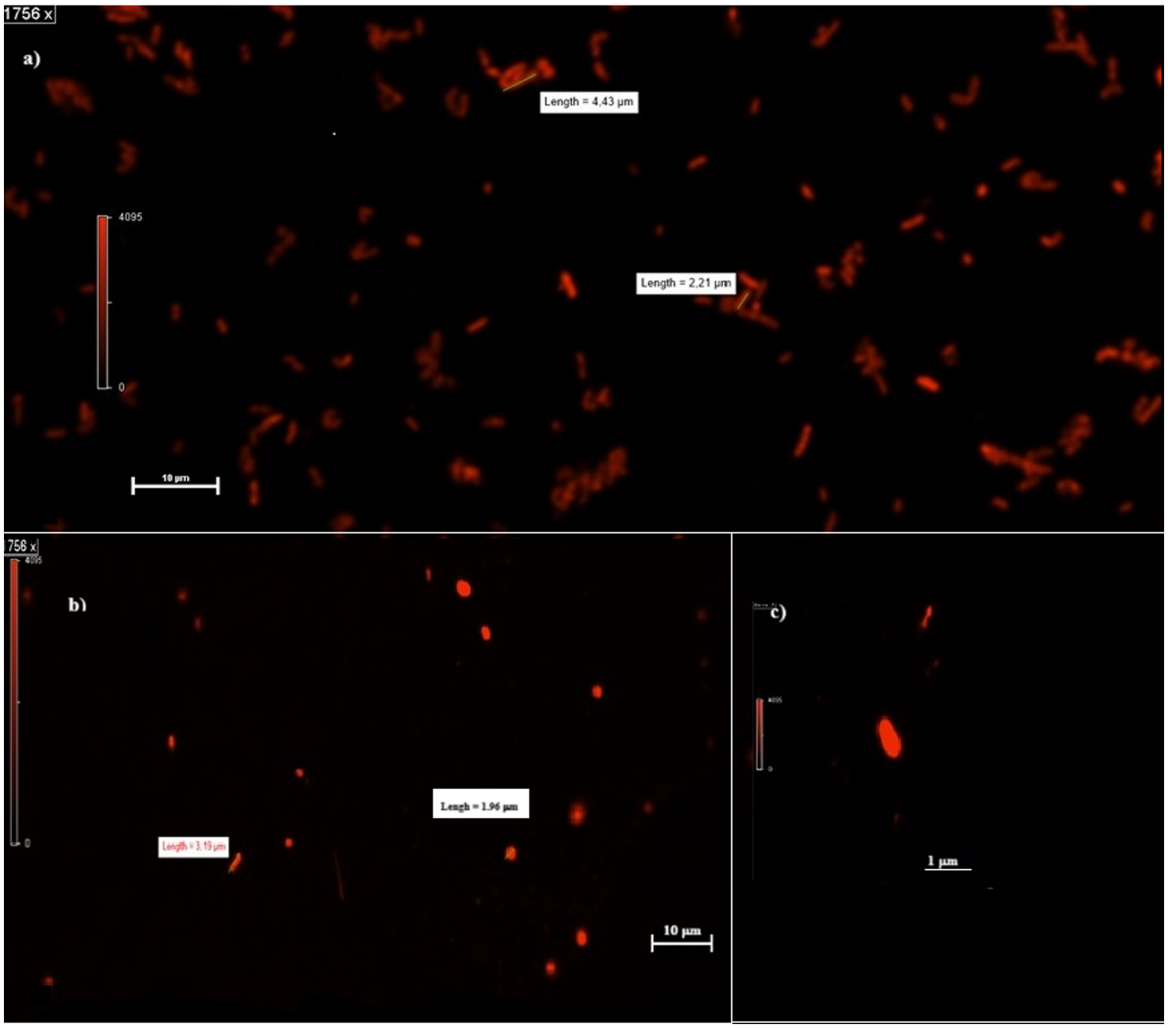
Planktonic cells in MFCs and MECs. **(A,B)** Shape and dimension of cells in MFCs **(A)** and MECs **(B)** catholytes. **(C)** MEC single-cell shape at higher magnification (3,512 x).

**Figure 3:**
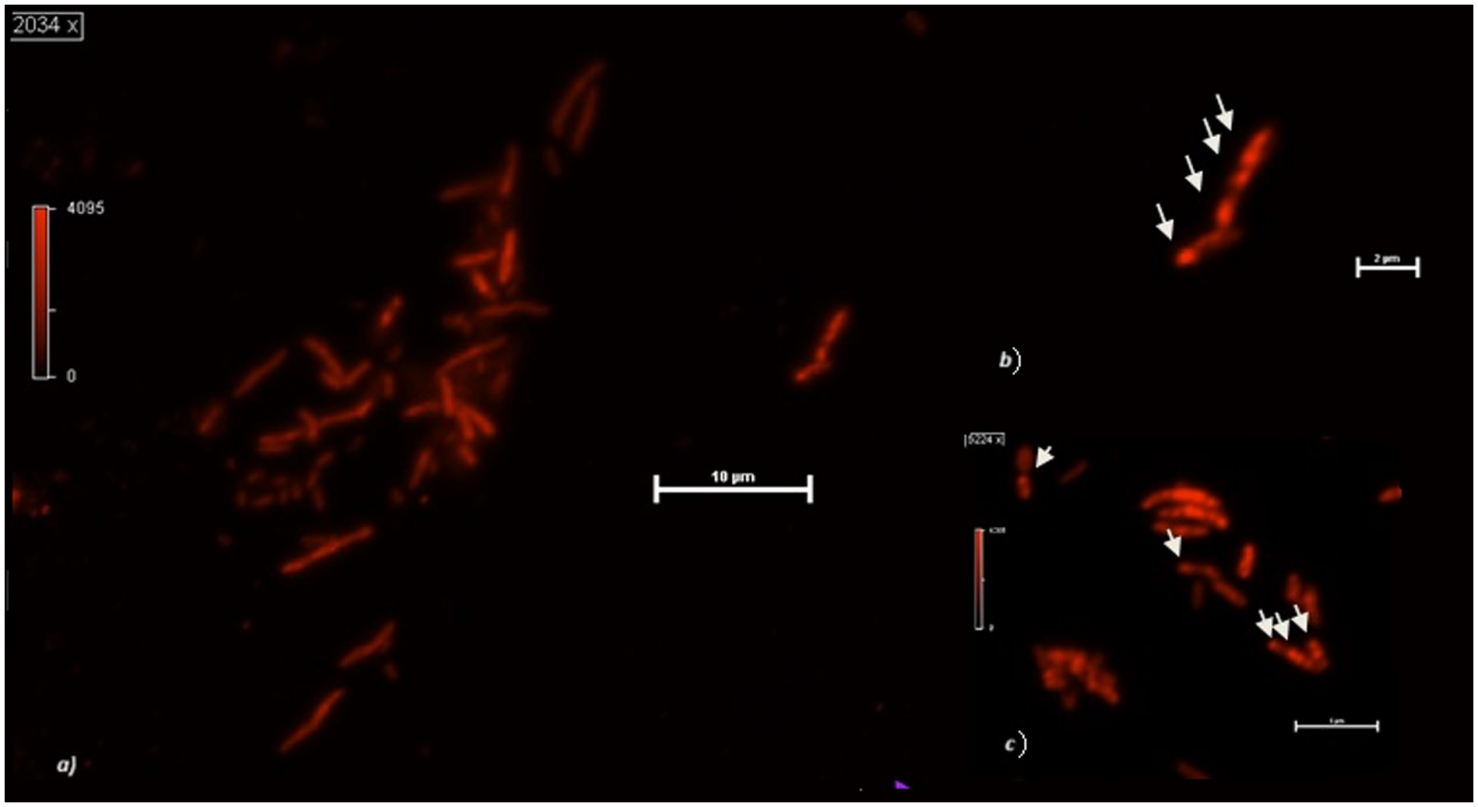
**(A)** *Cupriavidus necator* cells growing at the cathode of an MFC using 0.5% HCO_3_^−^. **(B)** Detail of two cells with PHB granules. **(C)** PHB granules in MFCs planktonic cells after 48 h operation with 0.5% HCO_3_^−^. The arrows indicate the intracellular granules.

When observed at the confocal microscope, cells from MECs revealed, in general, smaller size (between 2 and 3 μm in length) and more rounded morphology compared to the cells in MFCs and the control cultures (from 2 to up to 6 μm) (Figure 2). Further, the analysis of PHB granules’ intracellular distribution showed a tendency to their storage in the middle of the cell, although when present in a few numbers (1 or 2) they were in the cell periphery, near the cell poles, confirming what was previously reported by Vadlja et al. in 2016. Apparently, PHBs biosynthesis is associated with nucleoides because of a nucleoid-associated PhaM-PhaC1 complex, where PhaC1 is the PHB synthase (PhaC1) and PhaM is the granule-associated protein, with phasin properties, that can bind to PHB, DNA, and PhaC1 polymerase. The link between PhaC1 and chromosome DNA seems to be the basis of the non-random position of granules in the cell. Once formed, they undergo coalescence until occupying most of the cell volume if the growth conditions are favorable. Experimental evidence correlates the number and volume of granules in *C. necator* cells to nutritional factors such as carbon source and nitrogen availability. When grown in balanced nutritional conditions (i.e., in the presence of all nutrients present in proper amounts), cells contain no granules or in a number of one or two, with limited volume. With nutrient depletion, the shape of the cells starts changing, increasing their length, and the number and volume of granules increase as well, which is reported to be mostly in a number of 6. However, some authors report the presence of more than 10 granules per cell. The elongated cell forms are due to an interruption of cell division as no cell septum and nucleoid duplication are evident: how and why this happens is still to be clarified (Vadlja et al., 2016). The cells in MFCs catholyte appear to be in this metabolic state, with up to six granules which were present in most of the cells. The V_granules_/V_cell_ ratio was 74 ± 2% on the average in MFCs. With prolonged starvation, the number of granules decreases while increasing in volume until they occupy most of the cytoplasm, the cell decreases in size while becoming more rounded. This state is somehow like what is observed in most part of the cells grown in MECs. The image of the round-shaped and smaller cells at the confocal microscope was homogeneously fluorescent, without any relevant difference in the different areas of the cytoplasm, therefore we were not able to clearly detect any granules. When present (in a few long-shaped cells), they were in a number of 1 or 2, with a V_granules_/V_cell_ ratio of about 23 ± 3%. When observed at the confocal microscope, cells from control cultures were more cylindrical, with up to 4 granules per cell. The overall V_granules_/V_cell_ ratio was 40 ± 2% (Figure 3). A proper analysis at transmission electron microscopy (TEM) can reveal the detailed structure of *C. necator* cells grew in MECs, MFCs, and control cultures.

**Figure 4:**
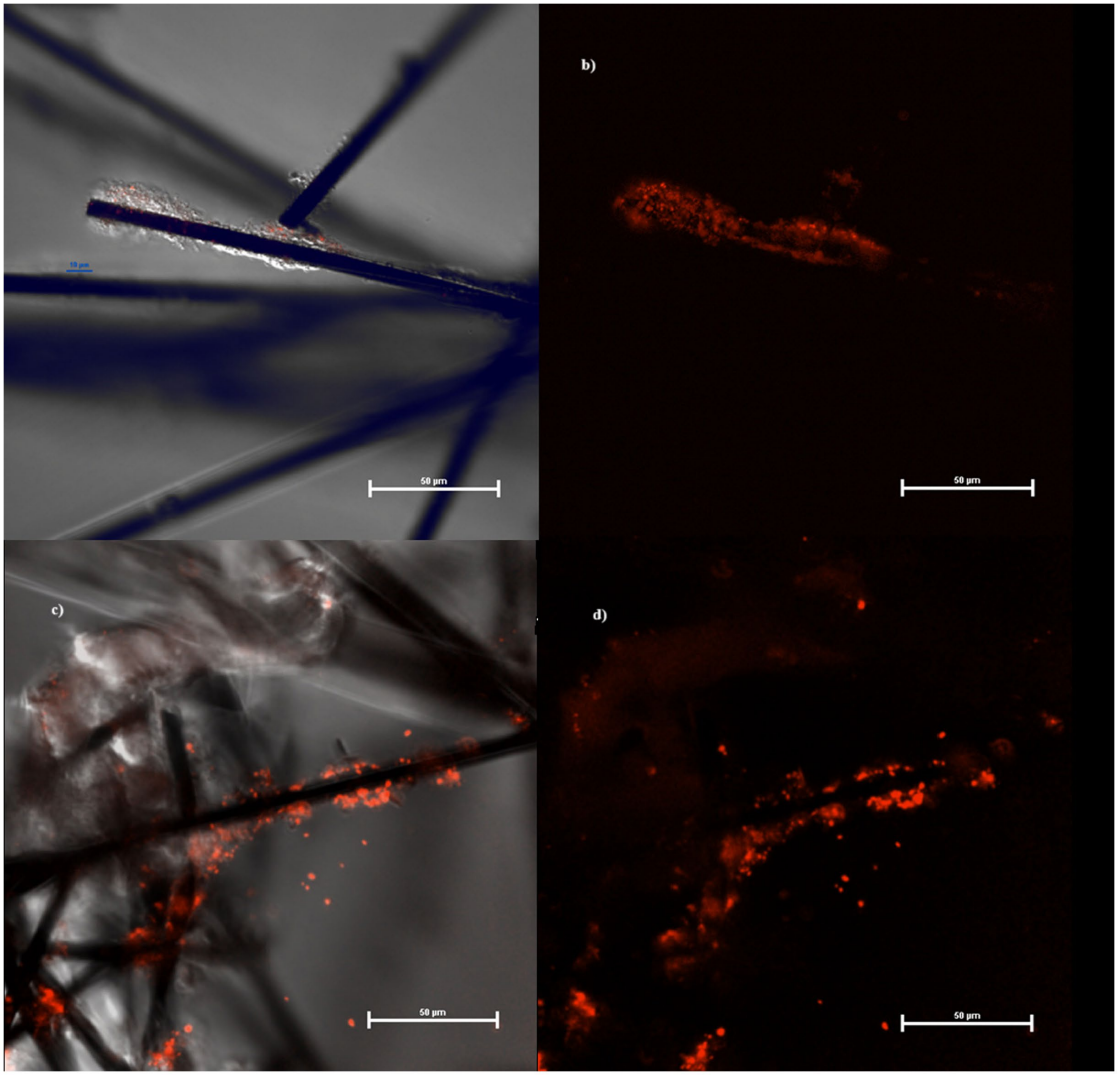
Biofilm at confocal microscope growing on MECs **(A,B)** and MFCs **(C,D)** fibers.

If compared with the control, MFCs stimulate granule biosynthesis while MECs did the opposite. According to some authors, the pronounced elongated cells (without clear evidence of septum formation) have stopped dividing for unknown reasons. Interestingly, we obtained these results using the same culture media, but exposing the cells to different redox potentials. Growth conditions were reflected also in biofilm structure and density in the cathodes of MECs and MFCs, with the latter colonized by a larger number of bacteria and a greater amount of biofilm. Further, because of the application of the external potential (and local production of H_2_?), cathode biofilm in MECs showed signs of detachment unlike what was observed in MFCs (Figure 4). Therefore, it is possible to assume that, in MECs planktonic cells were mainly involved in CO_2_ reductive assimilation while locally produced H_2_ mediates the electron transport. In MFCs, instead, biofilm is well developed and structured taking advantage of the direct electron transfer through the cathode.

### Metabolic analysis of *Cupriavidus necator*

**Figure 5:**
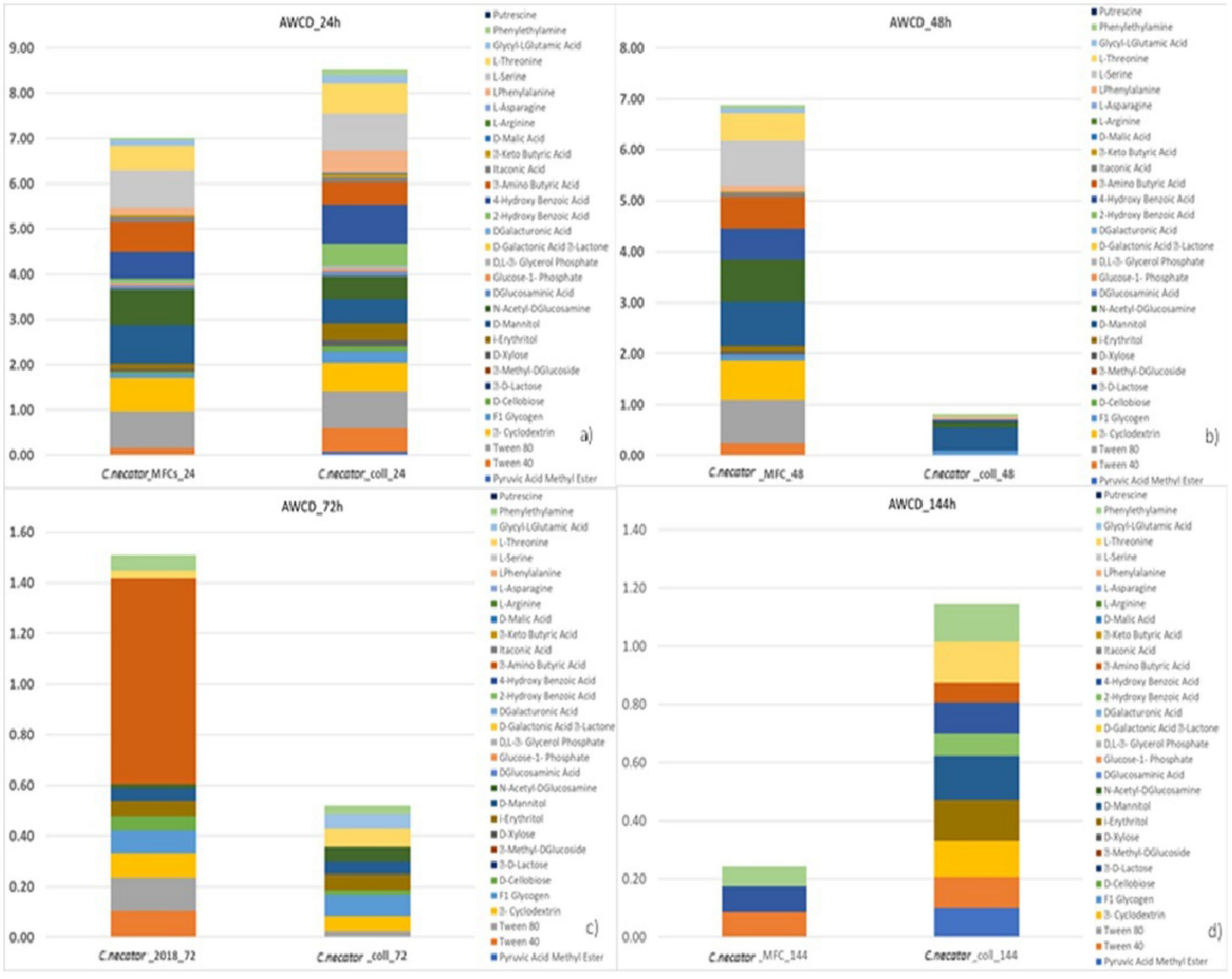
Metabolic profiling outcomes of *C. necator* strains. *C. necator*_MFC is the strain used in the ALE experiment and *C. necator*_coll is the strain never previously used in BESs.

When we tested the ability of the collection strain of *C. necator* to remove CO_2_ from the catholyte of a MFC, we measured just a 62% CO_2_ removal rate and 4 μg/mL of PHBs measured by the rapid throughput method, far below the 80% removal and 24 μg/mL in MFC with the strain previously used in former experimental activities. The results of AWCD for *C. necator*, showed that the metabolic activity of the reference strain (*C. necator*_coll) significantly decreased after 24 h of incubation, unlike what was observed in *C. necator*_MFC, (used in MFCs multiple times) showed an overall stable metabolic profile during the first 72 h (Figure 5). After 72 h at 30°C, the ability of both strains to use the organic carbon sources decreased, but the strain used in MFCs showed more activity linked to the utilization of L-Phenylalanine, Tween 80, *α*- cyclodextrin, D-mannitol, N-acetyl-D-glucosamine, L-serine, 4-hydroxy benzoic acid, and L-threonine. Nevertheless, after 144 h, the AWCD showed a dramatic decrease of the MFC strain with a metabolic activity limited to aromatic compounds such as Tween 40, 4-hydroxy benzoic acid, and phenylethylamine thus showing the residual activity of highly conserved metabolic pathways such as decarboxylation of l-phenylalanine, *β*-ketoadipate pathway and β -oxidation by esterase/lipase activity (Koseki et al., 2010; Nguyen, 2018; Luengo and Olivera, 2020; Weber et al., 2023). According to some authors, the activation of ancient metabolic pathways is particularly observed in the scarcity of nutrients as they require a lower amount of energy in terms of ATP (adenosintrifosphate). The consistent utilization of L-Phenylalanine could be ascribed to the decarboxylation of this amino acid, with CO_2_ release (that can be further used to produce PHBs) and phenylethylamine which is listed among the substrates able to still sustain *C. necator* growth after 144 h of incubation at 30°C. The improved use of amino acids like L-serine and L-threonine, L-aspartate as well as alternative carbon sources can be ascribed to N-scavenging activity particularly active during PHBs biosynthesis in nitrogen scarcity conditions (Pearcy et al., 2022). The deamination of glycine through the reverse Glycine Cycle (rGS), for example, leads to the production of acetyl-CoA, NADH, and reduced quinone without carbon loss through CO_2_ production (Westenberg and Peralta-Yahya, 2023).

On the other side, the increased utilization of Tween 80 and 40 as well as of aromatic compounds can be ascribed to the activation of alternative metabolic pathways for energy recovery. It has been demonstrated, for example, that *C. necator* can produce PHBs from aromatic compounds by using the 2,3-dioxygenase pathway, with benzoic acid as an intermediate (Berezina et al., 2015). Whether the improved utilization of the above-mentioned substrates is a result of a metabolic adaptation or a real adaptive evolution (i.e., a genomic modification) is still to be ascertained. In any case, according to the existing literature, our data seems to point to a link between the increased metabolic abilities and the improved biosynthesis of PHBs.

### Biosurfactant production

The drop collapse essay revealed the presence of surfactants in both MECs and MFCs catholytes, with MFCs drops collapsing immediately after being put under the microscope. This result is consistent with the *Pseudomonas/Shewanella* consortium being a strong surfactant producer (Gambino et al., 2017). The results of chemical analyses revealed the production of both anionic and non-ionic surfactants in the M9 medium supplemented with glycerol (Table 2). Strains that had never been used in BESs showed higher surfactant production compared to strains from previous studies. Nevertheless, the amount of energy produced (and overall stability) was higher in the BESs inoculated with the strains already used for several weeks in MFCs. We will discuss this result in the next section.

**Table 2:** Surfactants concentrations in MFCs and MECs.

Samples	Cationic (mg/L)	Anionic (mg/L)	Non Ionic (mg/L)
MFC	< 0.2	18.8	14.6
MEC	<0.2	20.7	14.9
MFC__coll	<0.2	27.1	27.4

The values are expressed as mg/L in catholytes. MFC and MEC strains underwent to the ALE experiment. MFC_coll was inoculated with strains (both at the anode and the cathode) never previously used in BESs.

### Electrochemical analyses

**Figure 6:**
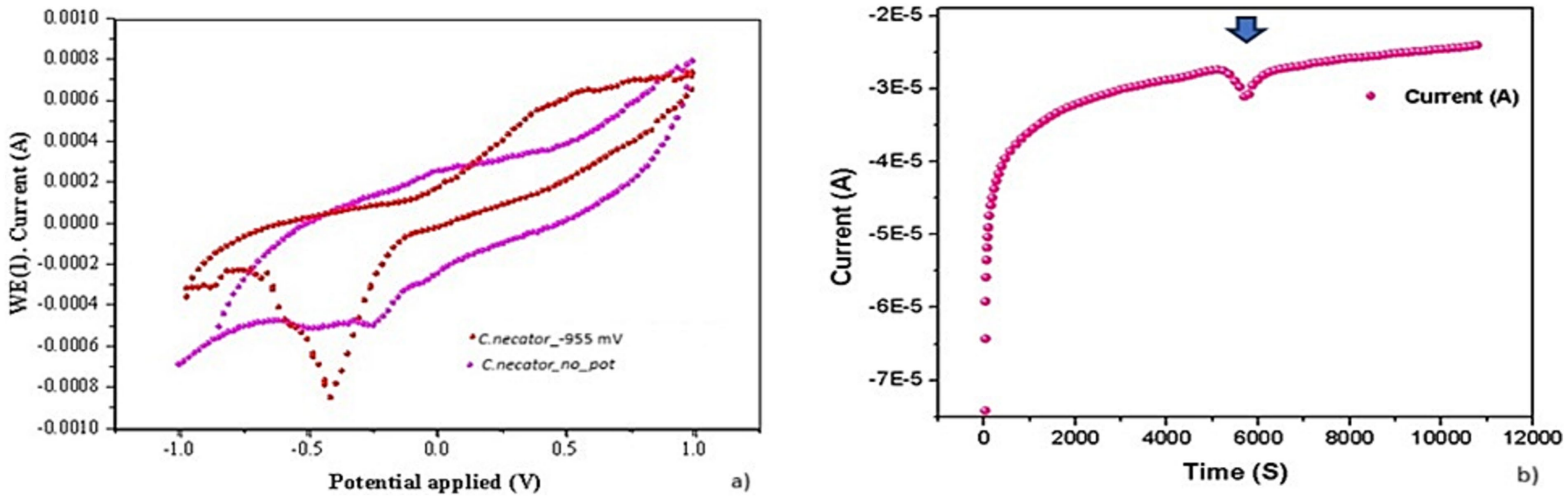
*Cupriavidus necator* DM428 biofilm after the application of −955 mV for 48 h (CV: 1 mV/s). **(A)** *C. necator* dm428 biofilm with no external potential applied (CV: −1 V: +1 V – 1 mV/s). *C. necator* dm428 sterile supernatant (0.22 mm pore filter) from +1,5 V MFC (CV: −1 V: +0 V – 1 mV/s). **(B)** Chronoamperometry of *C. necator* dm428 grown in the presence (4a) of −955 mV potential vs. Ag/AgCl (4a). The arrow indicates a peak of negative current appeared after 5,800 s.

Frielingsdorf et al. (2011) reported a study on a Group 1 hydrogenase, tolerant to oxygen unlike mostly hydrogenases already known, present in *C. necator* H16. Such a complex, formed by at least three metalloproteins with Ni-Fe, Fe-Fe, and Fe-S prosthetic groups, showed that it is actively involved in CO_2_ assimilation by splitting H_2_ into H^+^ and electrons. We know that the electrons from the H_2_ splitting are transferred to HCO_3_^−^ (the inorganic form of C that is assimilable by microorganisms), thus producing formic acid that enters the Calvin Benson Bessam cycle. The main product of this metabolic pathway is the glyceraldehyde-3-phosphate. This is, then, converted into acetyl-CoA, which is subsequently reduced to 3-hydroxybutyril-CoA, the monomer of PHBs (Humphreys and Minton, 2018). In 2016, Flanagan et al. published an interesting study about the electrochemical properties of Group 1 membrane-bound hydrogenases (MBH) in *C. necator*h16 by performing a CV on an enzyme-coated electrode. The results strongly suggest that the generation of a negative current is consistent with the occurrence of a biosynthetic process, (in their study the production of H_2_) as electrons are transferred from the cathode to the MBH complex. Further investigations revealed that, in MBH, the electrons produced from H_2_ degradation are transferred from the hydrogenase into a quinone pool, with the Ni-Fe MBH forming part of the bacterial respiratory chain involved in a direct transfer from the electrode. In our study, the prolonged application *to C. necator* of an external potential of −955 mV induced the formation of a visible cathode reduction peak, which was significantly reduced in CV performed on biofilm grown without any external energy input (Figure 5). A chronoamperometric analysis carried out at 10 mV/s revealed the appearance of the reduction peak, with a negative current peak of about 4 μA, after about 5,800 s, corresponding to 2.44 × 10^−7^ mol of electrons released from the cathode toward the biofilm. It is important to notice that, during the electrochemical tests, no hydrogen was provided. Our results are, therefore, consistent with those reported by Flanagan and Parkin (2016). However, the origin of the biosynthetic peak at about −370 mV (−165 mV *vs* Normal Hydrogen Electrode - NHE) in the biofilm grown under the application of –955 mV (−750 mV *vs* NHE) at the cathode and of the –280 mV (−75 mV *vs* NHE) peak in the biofilm formed in absence of applied potential is still to be ascertained. Nevertheless, these results are consistent with the involvement of the Mo-dependent formate dehydrogenase FdsDABG complex described by Harmer et al. (2023) who studied the redox potentials of the reaction centres of four subunits forming the heterotetrameric FdsDABG. According to the authors, electrochemical potentials with values between −130 and −210 mV *vs* NHE at pH 7.5 are consistent with the occurrence of two single-electron transfer steps, mediated by FMN and [4Fe−4S]2+ B6 co-factors. The meaning of the −75 mV (*vs* NHE) peak in biofilm grown in absence of external potential is still to be stated. *C. necator* expresses four types of hydrogenases encoded on the pHG1 megaplasmid that have prominent roles in autotrophic growth and ATP generation: SH, MBH, RH, (regulatory hydrogenase) are three catalytically active hydrogenases while little information are available about an actinobacterial hydrogenase (H_2_) that, given its slow H_2_ utilization rate (0.5 s^−1^) seems to play a role when H_2_ is present at low concentrations. However, recently it has been demonstrated how, in *C. necator* H16, redox chemistry is largely controlled by the soluble hydrogenase (SH) that creates reducing equivalents from the oxidation of H_2_ gas. This might be at the base of the different voltammograms obtained in MECs and MFCs (Figure 6). In a few words, it is possible that the different results obtained in the presence and absence of applied potential can be due to the influence that cathode redox potential has had on the activity of the pool of hydrogenases present in *C. necator*. The polarization experiments revealed good stability of MFCs inoculated with preformed biofilms and with the *C. necator* strain used several times in MFCs during previous experimental sessions.

**Figure 7:**
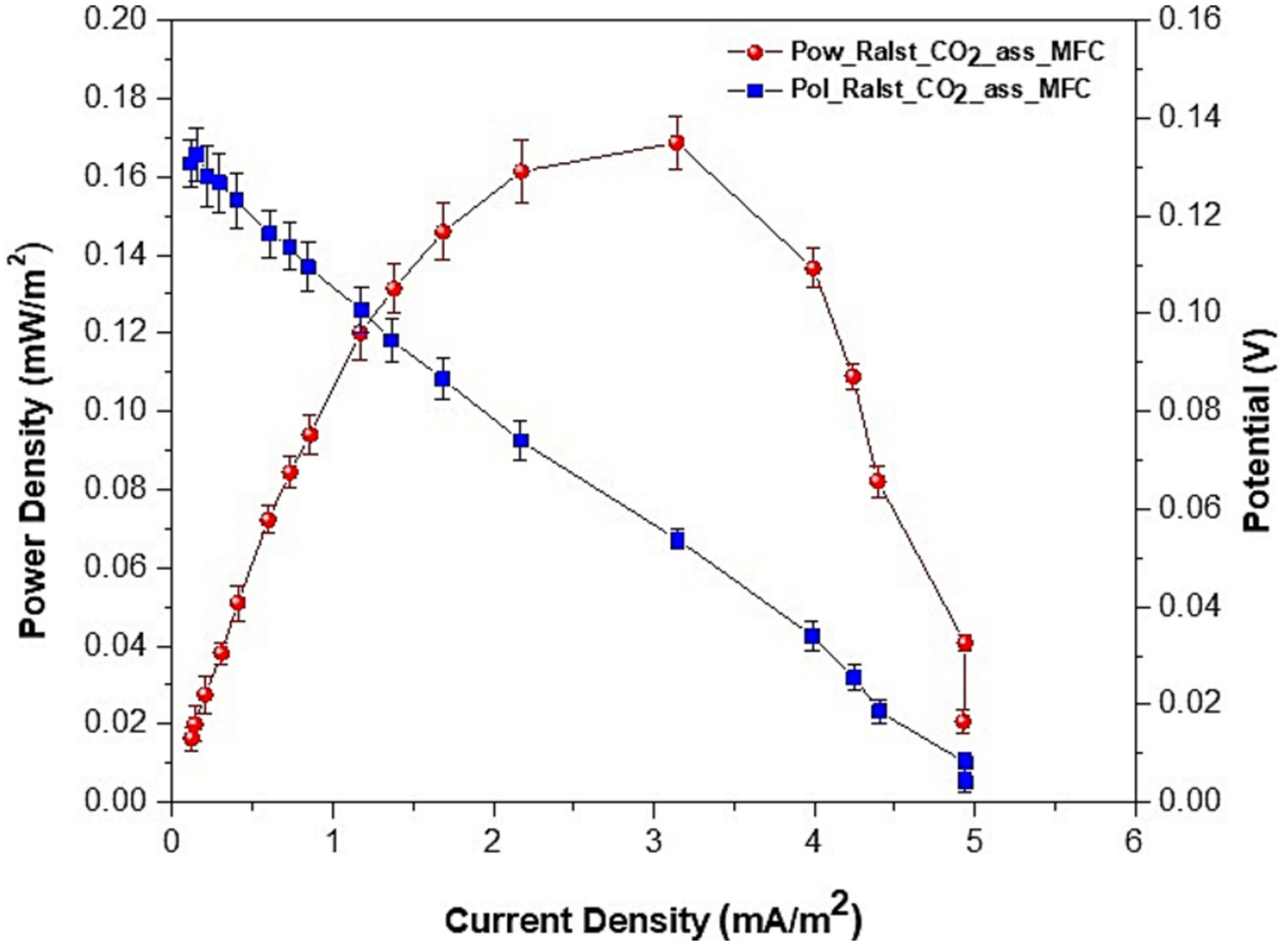
Polarization and power curves of MFCs: voltage, current and power densities average values ± SE of two series of experiments.

When we inoculated the strains directly in MFCs with sterile electrodes (MFC_no_biofilm) or used preformed bioelectrodes with bacteria from the collection culture (MFC_coll) we observed a voltage reversal after a few hours of operation and a lack of proper polarization. These last results seem to indicate a significant influence of bioelectrode preparation before their use in BESs (described in the materials and methods) as well as metabolic changes induced by the prolonged utilization of the strains in MFCs. During the CO_2_ capture experiment, all MFCs were connected to a 1,000 *Ω* external resistor: such value is used by many researchers during the start-up of MFCs to stimulate the electroactive bacteria at the anode. The polarization curves of MFC_1,000 Ω showed a maximum power of 0.169 ± 008 mW/m^2^, which was achieved when an external resistor of 2000 Ω was applied, while a maximum current of 4.9 ± 0.07 mA/m^2^ was achieved at 200 Ω (Figure 7).

Without a preformed biofilm, the MFC_no_biofilm did not polarize properly and started reverting at an external load of 22,000 Ω. When we used the DSMZ original strain and connected the MFC_coll to 1,000 Ω, we obtained an average current of 1.36 mA/m^2^ (lower than the 2.0 mA/m^2^ measured in the same conditions when using previously used isolates in MFC_no_biofilm) and a maximum power density of 0.153 mW/m^2^ at 18000 Ω. However, as previously said, also MFC_coll started reverting to lower values of applied external resistance and after being connected to the 1,000 Ω external resistor for less than 30 min, both MFC_no_biofim and MFC_coll switched to negative voltage values. Therefore, our results seem to confirm the important role of both biofilm maturity and cell metabolic adaptation in the stability of MFCs for CO_2_ biocapture.

The energy spent by the MFCs during the CO_2_ capture from the gas mix was 6.2 × 10^−2^ W.h/mol CO_2_ and 1.6 Wh/mol CO_2, respectively,_ in MFCs and MEC. Starting from the measure of current intensity provided at the cathode during the CO_2_ capture experiment, the moles of electrons supplied per mole of CO_2_ consumed in the catholyte by MFCs and MECs were 1.51E^−07^ and 4.46E^−04^ n*_e_*/mol_CO2_. Interestingly, the calculated amount of 1.51E^−07^ n*_e_*/mol_CO2_ is not far from the value measured during the chronamperometry after 5,800 s and equal to 2.44 × 10^−7^ mol as previously reported in this section. The first step toward CO_2_ reductive assimilation is its reaction with H_2_, locally formed in MEC, and H^+^ and electrons transferred from the anode compartment, in MFC. In all cases, CO_2_ reduction involves the Group 1 hydrogenase, starting from the n*_e_* supplied at the cathode of each system in the removal of one mole of CO_2_ we can estimate an overall amount of 2.23E^−04^ mol of H_2_ in MECs. In MFCs, we can not consider the direct synthesis of H_2_ produced as a consequence of water electrolysis, but the number of protons moving through the cation exchange membrane and directly being available together with electrons to the formate dehydrogenase whose amount has theoretically the same values of electrons provided by the anode: mole of H^+^ are therefore 1.51E^−07^ molH^+^/molCO_2_. It was impossible to estimate the amount of energy per mole of PHBs and surfactants produced as well as the Columbic efficiencies no information is available about the chemical structure of both by-products. We report in the supporting information file a table summarizing the results obtained in MFCs and MECs (Supplementary Table S2).

### Statistical analyses

**Figure 8:**
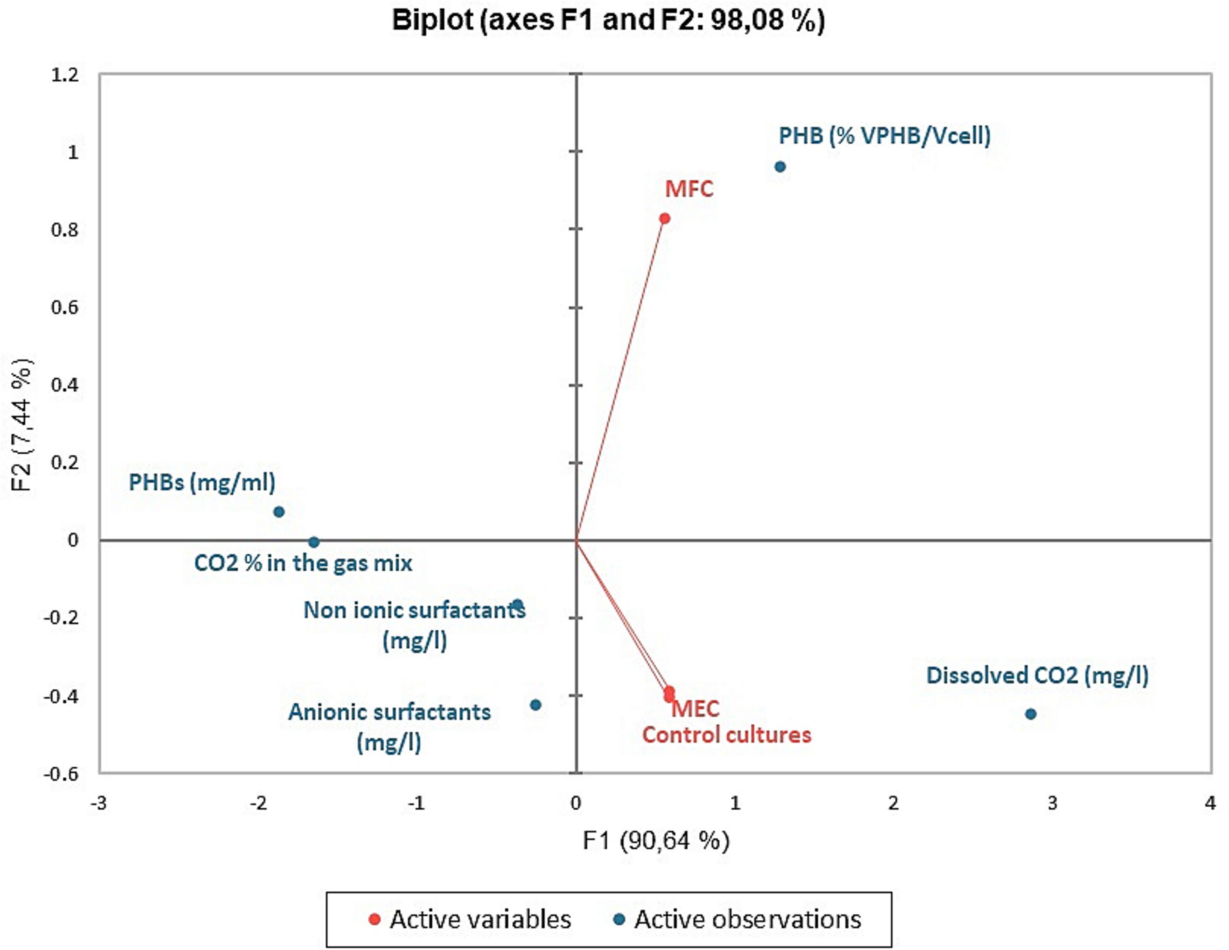
PCA analyses: biplot representing the active variables (MFCs, MECs, and control cultures) and the concentration of metabolites and CO_2_ in the media and its percentage in the gas mix.

**Figure 9:**
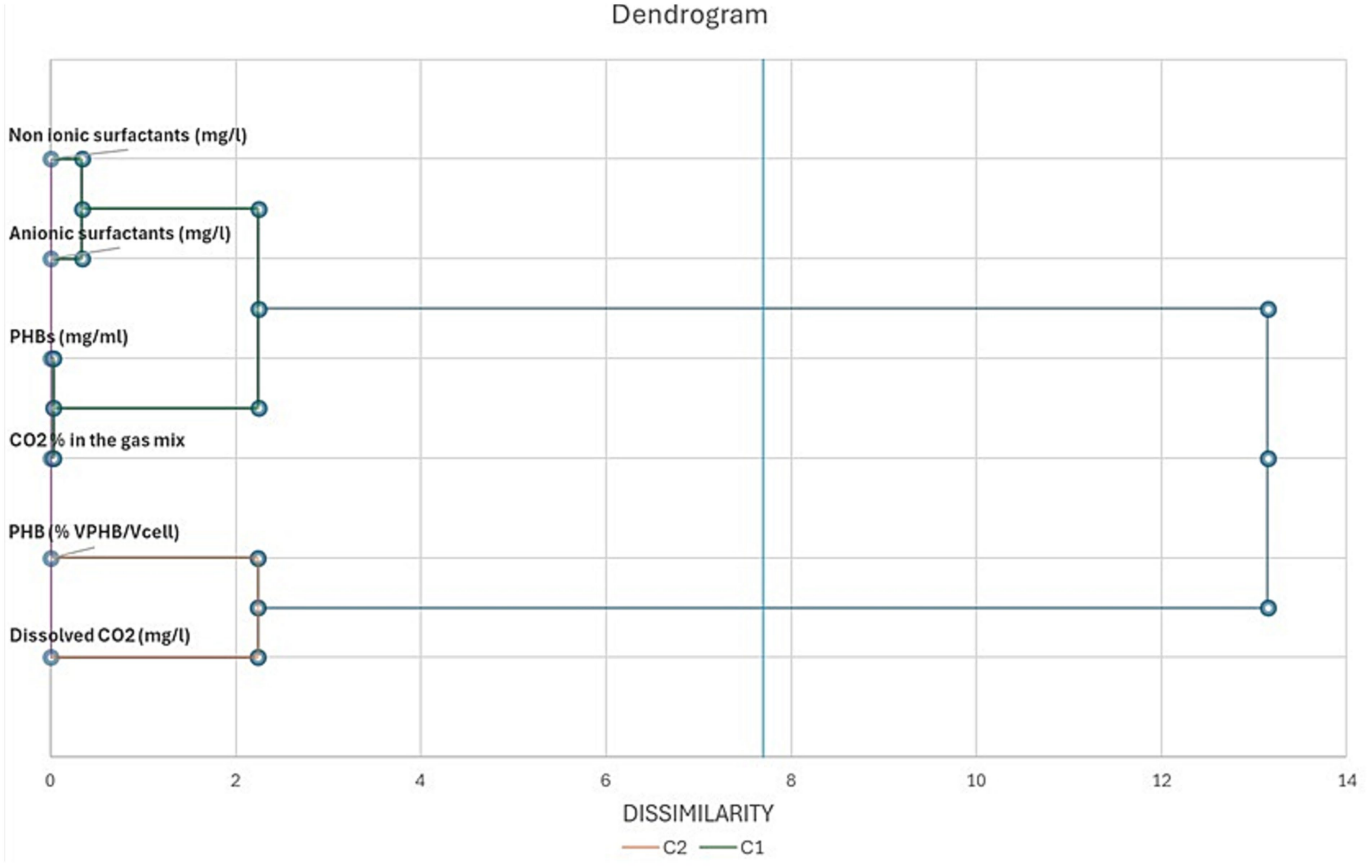
Dendrogram of metabolites, CO_2_ removal from the gas mix, PHBs granule volume and dissolved CO_2_ in *C. necator* growth medium. The parameters are arranged on the basis of their dissimilarity at *α* = 0.05.

In Figures 8, 9 we report the results of the PCA analysis and AHCA. Unlike what was expected, MEC and control cultures placed very close in the diagram, far distant from the MFCs, indicating that a higher correlation among the group of parameters was found between MECs and control cultures rather than with MFCs ones. Very likely, this result is mainly due to the low PHBs production in both MECs and control cultures. MFCs, instead, correlates very well with the accumulation of PHB granules in the cells (VPHB/Vcell). Biosurfactant concentration correlates with PHB concentration (mg/ml) and CO_2_ removal from the gas mix while they are negatively correlated with dissolved CO_2_ concentration, that well describes what happens in bioelectrochemical systems. The correlation among the different parameters is more evident in the cluster analysis, where biosurfactant are in the same cluster with PHB amount and CO_2_ concentration in gas mix while the volume of intracellular granules is statistically more correlated with dissolved CO_2_ in the DSMZ 81 growth medium.

## Practical implications

The co-production of biosurfactants and PHBs in MFCs offers a sustainable approach for industrial applications, integrating waste valorization with value-added bioproduct synthesis. One needs to address critical challenges to scale up this innovative bioelectrochemical technology. First, energy-efficient reactor designs are essential in order to optimize electron transfer, especially DIET, and maintain microbial activity deprived of excessive energy input. Modular, scalable MFC systems with efficient electrode materials and a high surface area can enhance productivity. Second, integrating CO₂ capture with biosurfactant and PHB synthesis requires precise control of redox conditions to balance metabolic pathways. Challenges include maintaining biofilm stability, ensuring consistent substrate availability, and managing downstream processing of PHBs and biosurfactants. Advances in electrode materials, such as conductive biochar or nanostructured carbon, can improve electron transfer and process efficiency. Coupling MFCs with renewable energy sources and continuous-flow systems can reduce energy demands, making the technology economically viable. Addressing these aspects could enable large-scale deployment for sustainable biomanufacturing.

## Conclusion

It is possible to produce PHBs and surfactants from, respectively, inorganic carbon and glycerol in MFCs inoculated with preformed, mature biofilms of *C. necator* at the cathode and *Pseudomonas aeruginosa/Shewanella oneidensis* consortium at the anode in a self-sustaining overall process, where a mechanism similar to direct electron transfer process established among the electrogenic consortium (at the anode) and electrotrophic microorganisms (at the cathode). Such a mechanism seems to establish when well mature biofilms, already accustomed to the nutritional conditions established in MFCs are present at the electrodes, providing also electrochemical stability to the system. The use of MFCs without preformed bioelectrodes as well as inoculated with strains not acclimatized to the conditions established in MFCs resulted in unstable systems. MECs were more efficient in removing CO_2_, from the gas mix although the amount of PHBs produced was lower than in MFCs. This result seems to indicate that, in MECs, the energy provided to the systems allowed the production of metabolites whose biosynthesis requires a higher amount of energy. Proper analyses of catholyte are needed to characterize the pool of metabolites produced by *C. necator* when grown at −955 mV cathode potential. A dedicated measurement system will be used to improve the monitoring configuration of the prototypes as future improvement as well. The preliminary results from confocal microscopy confirmed the trend obtained by the fluorescence measurements, but a quantitative analysis of PHBs produced in each system and the amount of energy spent by each system for their biosynthesis is needed, as well as the identity of the produced biosurfactants. In any case, our research showed that combining biosurfactants and PHB biosynthesis in MFCs is possible. The metabolic analyses on *C. necator* showed a prolonged metabolic activity as well as an increased ability to use molecules containing an aromatic ring such as *p*-hydroxybenzoic acid (PHBA), polysorbates (Tween 40) and aromatic amines, besides of aminoacids and polymers, as observed in a similar experimental activity in *C. saccharoperbutylacetonicum* NT-1. Further investigations, even at genomic level, are needed in order to evaluate the outcomes of the ALE used and the meaning of the metabolic changes observed in *C. necator*, the increased production of PHBs at the cathode and investigate the metabolic profiles of *P. aeruginosa* and *S. oneidensis* to further develop MFCs for CO_2_ capture and glycerol fermentation. Overall, this study presents a novel approach to simultaneously achieve CO_2_ capture, PHB production, and biosurfactant synthesis in MFCs, advancing the field of MET. The results demonstrate the enhanced metabolic activity of *C. necator*, with an increased ability to utilize aromatic compounds such as p-hydroxybenzoic acid and polysorbates. These findings offer a promising pathway for sustainable bioproduction. Further investigations, including genomic and metabolic profiling, are necessary to optimize MFCs for CO_2_ capture and glycerol fermentation in industrial applications.

## Data Availability

The raw data supporting the conclusions of this article will be made available by the authors, without undue reservation.
