# Supplementary material for: Bio-electrosynthesis of polyhydroxybutyrate and surfactants in microbial fuel cells: a preliminary study
Source: Front Microbiol. 2025 Feb 25;16:1372302. doi: 10.3389/fmicb.2025.1372302 (PMC11895702; doi:10.3389/fmicb.2025.1372302)
Supplement: Supplementary file 1 [file Data_Sheet_1.pdf]

SUPPORTING INFORMATION

S.1. Methodological approach to bioelectrodes formation

Bacteria can produce biofilms on both biotic and abiotic surfaces as a physiological activity of great part of microorganisms. Biofilm formation can be enhanced by applying dynamic (with medium flowing), and even semi-dynamic conditions (i.e in shaking conditions), able to foster the biosynthesis of a more robust EPS-matrix, the availability of nutrients for growth, and to promote the expression of molecules involved in signal transduction, as demonstrated in *P. aeruginosa* [1][2] This results in the generation of denser and thicker biofilms able to resist environmental stressors, even in multispecies biofilms [1]. Nevertheless, its properties can change according to the environmental conditions in which microorganisms grow, and according to the wettability and structure of the surface to be colonized, the density of the starting microbial culture and medium composition, charges on microbial cell as well as on the substratum surface, hydrophobic and electrostatic interactions [3]. In bioelectrochemical systems, the availability of a conductive surface (carbon cloth, for example) makes the electrode an electron sink but also helps in enriching the electrochemically active bacteria by promoting adherence, therefore, electroactive bacteria using a direct electron transfer mechanism (conductive piles as in *S.oneidensis*) are the first ones to colonize the surface, while bacteria using indirect electron transfer (like electron shuttles as in *P.aeruginosa*) are less keen to directly colonize the surface, being at the interface with the medium or growing as planktonic cells. [3]. While producing the biocathodes, we avoided the use of gaseous CO_2 as the presence of bubbles in the medium might have affected the microbial adhesion to the electrode surface. Therefore, we added to the DSM 81 medium $NaHCO_3$ (0.5%), which resulted in a CO_2 concentration of 28±2 mg/L, measured with Mettler Toledo CO_2 connected to M400 transmitter (Mettler Toledo)

CO_2 flow rate calculations

Microbial metabolism is affected by the availability of nutrients and their concentration over time. When nutrients are provided in the form of gas, the flow rate is an important parameter as it refers to the rate at which gases, such as CO_2, are introduced into the culture vessel to maintain the desired conditions for microbial growth. When CO_2 is used in microbial culture, the gas flow rate becomes especially important as it can impact various aspects of the culture, such as pH regulation, dissolved gas concentration, and overall growth performance of the microorganisms. The gas flow rate is the volume of gas (in this case, CO_2) that is delivered per unit of time into the microbial culture vessel. The experiment was carried out at 20°C, therefore we referred all calculations to this temperature value.

First, we considered the mass conservation law for incompressible gases [1]:

$$\rho vA=K \quad [1]$$

Where ρ is the gas density at 20°C, v is the speed of the gas through the pipe and A is the area of the section of the pipe. This means that gas speed is a function of the pipe section, being the density a constant. We, then, used the Bernoulli equation to calculate firstly the pressure of CO_2 at the exit of the needle used to sparge the gas mix in catholytes and control cultures and, then the speed of the flow. Bernoulli equation is, in fact, a correlation from the conservation equations to demonstrate a relation between velocity, elevation, and pressure in a nonviscous (frictionless) fluid. Formula 2 represents the general form of the Bernoulli equation:

$$\frac{1}{2}\rho V^2 + P + \rho gz = \text{constant} \quad (2)$$

Or if we consider the gas flow between two different points of a pipe, the equation becomes

$$P_1 + \frac{1}{2}\rho v_1^2 + \rho g z_1 = P_2 + \frac{1}{2}\rho v_2^2 + \rho g z_2 \quad (3)$$

Where V is the velocity of the gas, ρ is its density, P_1 and P_2 are the pressure at the two points, g is the gravity force, and z is the elevation between the outlet of the gas cylinder from the dispersing points (i.e. the bottoms of the cathode chambers and culture vessels). According to our calculations, the speed V_2 at the dispersion point is 3.71 m/s. We calculated the volumetric load of CO_2 flowing through the pipe according to the following equation (4):

$$Qv = A*v*t / t = A*v \quad (4)$$

Where Qv is the volumetric load, A is the inner section of the needle (0.821 mm), v is the gas speed and t are the time expressed as seconds. Qv is therefore $1.93*10^{-6}$ m^3/s

We then estimated the gas mix mass flow according to (5) being 1.61 kg/m^3 the overall density of the gas mix (68%N_2, 20% O_2, 10%CO_2, 2%H_2 at 20°C) (ρ)

$$\dot{m} = Q \backslash \rho \quad (5)$$

m is, then, 3.10 $*10^{-6}$ kg/s. As CO_2 represented 10% of the overall mass, we considered a mass flow (mCO_2) of $3.10*10^{-7}$ kg/s. Therefore, we converted the mass into moles, having a flow of 7.06 * 10^{-6} mol/s which is 4.23 * 10^{-4} mol/min. By applying the ideal gas law equation, we calculated the volume of CO_2 provided every minute.

Henry's Law calculations for CO_2

In order to calculate the concentration of CO_2 at saturation in the catholyte, we applied Henry's law equation for gas dissolution in water applicable, with good approximation, to dilute water solutions (with M<1). According to Henry's La, the concentration of CO_2 in water is expressed by:

$$C_{CO2} = \boldsymbol{P}_{CO2} * \boldsymbol{K'}$$

where C_{CO2} is the concentration of CO_2 in a solvent (in this case, water) and expressed as mol/L, K' is Henry's constant for CO_2 dissolved in water at 20°C and expressed as mol/L atm, and P is the partial pressure of the gas on the solvent at saturation, expressed in atm. As we used a gas mix composed of 68% of N_2, 10% of CO_2, 20% O_2 and 2% H_2 we used Dalton's law to calculate the partial pressure of CO_2 in the mix, being the pressure of the mix equal to 1.2 atm, P_{CO2} was 0.12 atm.

Additional tables and figures

Table 1S: Criteria for surfactant producers' classification.

Collapse time (s)	Score	Classification
>60	-	No producer
45-60	+	Very weak producer
30-45	++	Weak producer
15-30	+++	Medium producer

0-15	++++	Strong producer

Table 2S: Overall performance of MECs and MFCs in terms of CO2 capture and utilization

	**MFC**	**MEC**
CO_2 % in the gas mix*	1.9±0.3	1.4±0.2
Dissolved** CO_2 (mg/l)	65.4± 2.6	45.6 ±1.8
Dissolved (CO_2) mMol/l	1.48±0.06	1.0±0.04
PHBs (mg/ml)	0.028 ± 4.0E-03	0.0122±.2E-03 mg/ml
PHB (% V_{PHB}/V_{cell})	74±2%	23±3%
Anionic surfactants (mg/l)	14.6	14.9
Non ionic surfactants (mg/l)	18.8	20.7

References

1) Nastro, R.A., Arguelles-Arias, A., Ongena, M. et al. Antimicrobial Activity of Bacillus amyloliquefaciens ANT1 Toward Pathogenic Bacteria and Mold: Effects on Biofilm Formation. Probiotics & Antimicro. Prot. 5, 252–258 (2013). https://doi.org/10.1007/s12602-013-9143-1 2.
2) Zheng S, Bawazir M, Dhall A, Kim H-E, He L, Heo J and Hwang G (2021) Implication of Surface Properties, Bacterial Mo-tility, and Hydrodynamic Conditions on Bacterial Surface Sensing and Their Initial Adhesion. Front. Bioeng. Biotechnol. 9:643722. doi: 10.3389/fbioe.2021.643722
3) Arunasri K, Venkata Mohan S., (2019). Chapter 2.3 - Biofilms: Microbial Life on the Electrode Surface, Editor(s): S. Venkata Mohan, Sunita Varjani, Ashok Pandey,In Biomass, Biofuels and Biochemicals, Microbial Electrochemical Technology, Elsevier, 2019, Pages 295-313, ISBN 9780444640529, https://doi.org/10.1016/B978-0-444-64052-9.00011-X.
4) Tarraran L., Bozzolo Lueckel F., Tommasi T., Contador F.I.S, Fino D. (2022). A practical method for gas changing time estimation using a simple gas-liquid mass transfer model, Journal of Microbiological Methods, 200, 106544, ISSN 0167-7012, https://doi.org/10.1016/j.mimet.2022.106544
